# First-principles experimental demonstration of ferroelectricity in a thermotropic nematic liquid crystal: Polar domains and striking electro-optics

**DOI:** 10.1073/pnas.2002290117

**Published:** 2020-06-10

**Authors:** Xi Chen, Eva Korblova, Dengpan Dong, Xiaoyu Wei, Renfan Shao, Leo Radzihovsky, Matthew A. Glaser, Joseph E. Maclennan, Dmitry Bedrov, David M. Walba, Noel A. Clark

**Affiliations:** ^a^Department of Physics, University of Colorado, Boulder, CO 80309;; ^b^Soft Materials Research Center, University of Colorado, Boulder, CO 80309;; ^c^Department of Chemistry, University of Colorado, Boulder, CO 80309;; ^d^Department of Materials Science and Engineering, University of Utah, Salt Lake City, UT 84112

**Keywords:** liquid crystal, ferroelectric, nematic, polar, liquid

## Abstract

We report the experimental determination of the structure and response to applied electric field of the lower-temperature nematic phase of the previously reported calamitic compound 4-[(4-nitrophenoxy)carbonyl]phenyl2,4-dimethoxybenzoate (RM734). We exploit its electro-optics to visualize the appearance, in the absence of applied field, of a permanent electric polarization density, manifested as a spontaneously broken symmetry in distinct domains of opposite polar orientation. Polarization reversal is mediated by field-induced domain wall movement, making this phase ferroelectric, a 3D uniaxial nematic having a spontaneous, reorientable polarization locally parallel to the director. This polarization density saturates at a low temperature value of ∼6 µC/cm^2^, the largest ever measured for a fluid or glassy material. This polarization is comparable to that of solid state ferroelectrics and is close to the average value obtained by assuming perfect, polar alignment of molecular dipoles in the nematic. We find a host of spectacular optical and hydrodynamic effects driven by ultralow applied field (E ∼ 1 V/cm), produced by the coupling of the large polarization to nematic birefringence and flow. Electrostatic self-interaction of the polarization charge renders the transition from the nematic phase mean field-like and weakly first order and controls the director field structure of the ferroelectric phase. Atomistic molecular dynamics simulation reveals short-range polar molecular interactions that favor ferroelectric ordering, including a tendency for head-to-tail association into polar, chain-like assemblies having polar lateral correlations. These results indicate a significant potential for transformative, new nematic physics, chemistry, and applications based on the enhanced understanding, development, and exploitation of molecular electrostatic interaction.

## Significance

Conspicuously in the background in the history of liquid crystals is the ferroelectric nematic (N_F_) phase. Predicted by Debye and Born 100 y ago and since revisited extensively, in systems ranging from colloidal suspensions of rods or discs to melts of polar molecules, the existence of the N_F_ has never been certain, and it has never emerged in interest or applicability from the shadow of its familiar cousin, the dielectric nematic, the key component of the displays that enabled the portable computing revolution of the 20th century. Here we show, in a previously reported thermotropic material, defining evidence for ferroelectricity and a host of emergent polar behaviors that promise to remake the science and technology of nematics.

The first theoretical treatments of collective molecular orientation in liquids, by Debye (1) and Born (2), were electrostatic versions of the Langevin–Weiss model of the paramagnetic/ferromagnetic transition in solids (3). Born envisioned the orientational ordering of rod-shaped molecules of a nematic as a phase transition, the proposed ordering mechanism being the interaction of molecular electric dipoles, so that the resulting nematic phase was ferroelectric, i.e., predicted to have a spontaneous nonzero polarization density. Thus, the notion of LCs with polar order, introduced more than a century ago, has grown as a field of broad interest and challenge at the frontiers of LC science, stimulating rich themes of novel chemistry and physics (4–13)

However, following Born’s model, some calamitic molecules without molecular dipoles were found to exhibit nematic phases (14), while ferroelectricity failed to materialize as a molecular nematic phenomenon. Born’s calculation thus appeared to be incomplete, stimulating a variety of different models of nematic ordering in which both steric and/or electrostatic interactions were considered. These included the Maier–Saupe theory (15), where steric interactions produced apolar (quadrupolar) order, and others in which the nematic ordering could also be polar (4, 5, 16–22). The appearance of polar ordering in these models and Born’s is considered to be an equilibrium transition between bulk phases of higher and lower symmetry (23, 24). The models propose order parameters constructed to characterize this change of symmetry, and provide benchmarks for experimental testing, predicting pretransitional behavior as the phase transition is approached from higher or lower temperature, as well as describing the properties of the polar ordered phase and its distinct symmetry-related states. In the case of relevance here, of a uniaxial, nonpolar nematic transitioning to a uniaxial, polar nematic with the polarization along the director, there are two ordered states related by reflection through a plane normal to the polarization. If such states coexist in a sample, they must form reflection-related domains with opposite polarization separated by well-defined domain walls (25, 26, 17). Such polar domains and their boundaries are also described by the models, specifically by the elasticity and order parameter energetics of the polar phase, making the domains the signature features of spontaneous polar ordering to be probed and understood in characterizing the nature of the phase transition. If such domain boundaries can be moved or removed by application of a field, then the mean polarization can be changed, and if this motion is irreversible, then the polar phase will exhibit switching and hysteresis as emergent properties and can be considered macroscopically ferroelectric (27). Here we present the direct observation of such spontaneously broken symmetry in the form of domains of opposite polarization, grown without applied electric field, as a first-principles demonstration of ferroelectricity in a thermotropic, uniaxial, nematic liquid crystal (LC) of rod-shaped molecules.

**Fig. 1.**
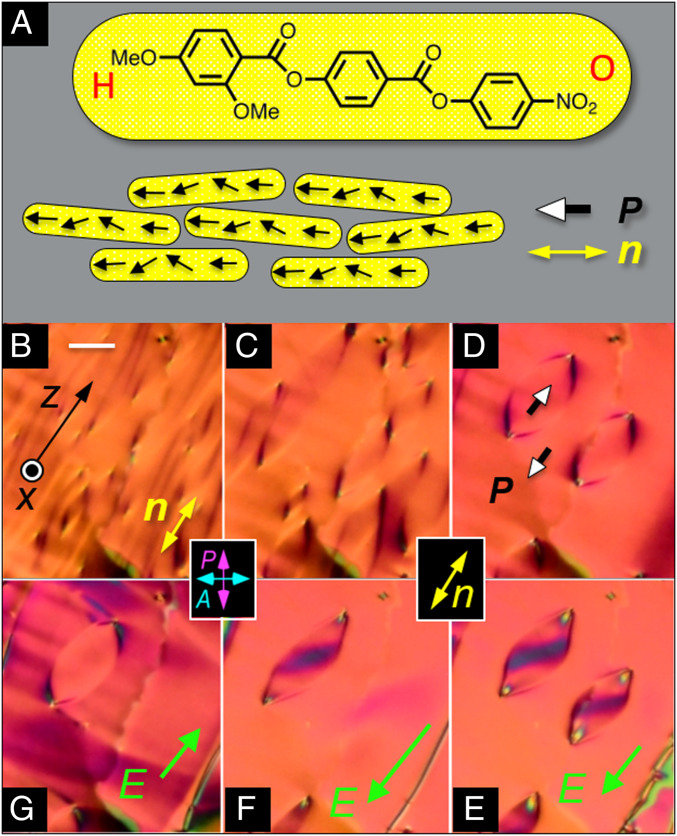
Ferroelectric nematic phase. (*A*) Structure of RM734 and schematic of molecular alignment in the ferroelectric nematic (N_F_) phase. The molecular organization is translationally symmetric in 3D and macroscopically uniaxial, with local mean molecular long axis, ***n***(***r***), aligned generally along the buffing direction ***z***, and polar, with a local, mean molecular dipole orientation, ***P***(***r***), along ***n***. H and O are used to represent the methoxy and nitro ends of the molecule, respectively. (*B*–*G*) DTLM images showing electro-optic evidence for ferroelectricity in a planar-aligned cell of RM734 in the N_F_ phase (*t* = 11 µm thick). In the higher-temperature N phase, *P*(***r***) = 0, but when cooled into the N_F_ phase without an applied field, RM734 spontaneously forms macroscopic domains with *P* > 0 or *P* < 0. When slowly cooled below the N_F_ phase transition at *T* = 133 °C, the initial texture (*B*) coarsens into a pattern of domains with distinct boundaries (*C*). (*D*–*G*) *T* = 120 °C. Starting from *D* with no field, application of an ultrasmall in-plane test field |*E*_z_| ∼0.5 V/cm along the buffing direction produces reversible reorientation of ***P*** without changing its magnitude. (*E* and *F*) Application of a negative *E*_z_ starts the in-plane reorientation of ***n***(***r***) about ***x*** inside the domains, producing the dark bands there, while (*G*) positive *E*_z_ produces reorientation outside of the domains, proving that these regions are of opposite polarization. The *E ∼* 1 V/cm threshold field for this reorientation indicates that ***n***(***r***) in these domains is coupled to ***E*** by a polarization *P* ∼5 µC/cm^2^, which is comparable to the bulk polarization density measured electronically. The higher applied field in *F* has moved the boundary of one lenticular domain to increase the area with the field-preferred orientation, effecting a hysteretic reversal of ***P***(***r***). (Scale bar, 30 µm.)

In 2017, Mandle et al. (28) and Kikuchi et al. (29) separately reported new LC compounds exhibiting unusual phase behavior: two distinct, fluid nematic phases separated in temperature by a weakly first-order phase transition. In both cases, the molecules were rod-shaped, with several intramolecular dipoles distributed along their length whose projections onto the molecular long axis summed to a large overall axial dipole moment of ∼10 Debye. The high-temperature phase of both mesogens was reported to be a typical nematic, but they exhibited dramatic paraelectric (30, 29) and ferroelastic (30) pretransitional effects, with a dielectric constant surpassing 1,000 as the transition to the low-temperature phase was approached. The low-temperature phase exhibited enhanced dipolar molecular associations, reported to be antiparallel in the Mandle system (28) and suggested to be parallel in the Kikuchi system, the latter being termed “ferroelectric-like” (29), giving macroscopic polar ordering in response to an applied electric field. Mandle et al. subsequently synthesized a number of homologs of their molecule in an effort to develop structure–property relationships for this phase (31) and pursued, in collaboration with the Ljubljana group, a series of physical studies on one of these (RM734), shown in Fig. 1*A*, leading to the claim that this phase was locally polar, as evidenced by second harmonic generation, but, on some longer scale, an antiferroelecric splay nematic (32, 33, 30, 34, 35), a modulated phase stabilized by local director splay, of the type originally proposed by Hinshaw et al. (36).

Our resynthesis of RM734 (*SI Appendix*, section S1) and observation of its electro-optic behavior using polarized light microscopy provides no evidence for a splay nematic phase but rather leads us to the unambiguous conclusion that upon cooling from the higher-temperature, nonpolar, uniaxial nematic (N) phase, RM734 undergoes a transition to another uniaxial nematic (N_F_) phase that is ferroelectric. The key evidence for this result is the first observation in a nematic LC of the defining characteristics of ferroelectricity: 1) the formation, in the absence of applied electric field, of spontaneously polar domains of opposite sign of polarization separated by distinct domain boundaries and 2) field-induced polarization reversal mediated by movement of these domain boundaries, as summarized in Fig. 1.

In the N phase, the local texture of the planar-aligned cell shown in Fig. 1 is optically featureless. On cooling toward the N_F_ phase, a random pattern of stripes extended along the buffing direction appears. Once in the N_F_ phase, these stripes coarsen, leading to a texture that is again local optically featureless (Fig. 1 *B*–*D*) but characterized on a larger scale by a pattern of well-defined lines, some delineating distinct, lens-shaped domains 100 µm or more in extent (Fig. 1 *D*–*G*), all formed in the absence of applied electric field. Application of an ultrasmall (∼1 V/cm), in-plane, DC test field, ***E***, applied along ***z***, parallel to the in-plane buffing and therefore to the director ***n***(***r***), shows that for *E* > 0 the director inside these domains begins to reorient, while the orientation outside remains fixed (Fig. 1*E*), whereas for *E* < 0 the region outside the lens-shaped domains reorients, and the orientation inside remains fixed (Fig. 1*G*), indicating that the domain boundaries separate regions with opposite response to in-plane field and therefore of opposite in-plane polarization. The lack of response to increasing *E* outside of the lenses in Fig. 1*E* and inside the lens in Fig. 1*G* shows that ***P*** and ***n*** are colinear in these domains in the field-free condition. Increasing the field causes the domain boundaries to unpin, shrink, and disappear (Fig. 1*F*), moving hysteretically to increase the area of the field-preferred state. The observations of Fig. 1, constituting a first-principles demonstration of nematic ferroelectricity, are described in more detail below and in *SI Appendix*, sections S3 and S4.

## Results and Discussion

### Electro-Optic Observation of Planar-Aligned Cells.

Depolarized transmission light microscopy (DTLM) observations of RM734 were made principally in cells with a *t* = 11-µm-wide gap between the glass plates, one of which was coated with a pair of planar ITO electrodes uniformly spaced by *d* = 1.04 mm, which enabled application of an in-plane electric field, ***E***, between them, largely parallel to the cell plane (*y*, *z*). The plates were treated with a polyimide layer buffed in the ***z*** direction, normal to the electrode edges, so that the applied field was along the buffing direction: ***E*** = ***z****E* (*SI Appendix*, Fig. S3). The cells were filled by capillarity with the LC in the isotropic phase. Both the N and N_F_ phases were studied, with results as follows.

### Nematic Phase.

When cooled into the nematic (N) phase, RM734 formed textures with the nematic director, ***n***(***r***), the local mean molecular long axis and the optic axis, parallel to the plates (planar alignment). The white-light birefringence color at *T* = 140 °C was a uniform pale yellow-orange, in the third-order Michel–Levy band (retardance ∼ 1,500 nm) (37), with the larger index for optical polarization along ***n***. The azimuthal orientation of ***n***(***r***) was generally along ***z*** but with a fixed pattern of in-plane orientational defects and weak, continuous variations of the in-plane orientation, as seen in *SI Appendix*, Figs. S4 and S6*A*, suggestive of a relatively weak coupling to the azimuthal anisotropy of the surface. Measurements give a uniaxial birefringence Δ*n* ∼ 0.2 (*SI Appendix*, Fig. S17), suggesting that the alignment is planar, with ***n***(***r***) nearly parallel to the plane of the plates. Tilting the cell away from being normal to the light beam did not reveal significant tilt of ***n***(***r***) out of the cell plane, but a small (few-degree) pretilt may be present. In addition to the locally uniform, planar texture imposed by the surfaces upon cooling into the N phase, we observed a few twisted areas (*SI Appendix*, Fig. S6*A*), but generally, the local preferred orientation was the same on the two plates and therefore likely established by a combination of the buffing with surface memory (38) of the nematic director pattern as it was first growing out of the isotropic.

### Ferroelectric Nematic Phase.

Upon cooling through the N–N_F_ transition, the cell becomes patterned with a texture of irregular domains extended locally parallel to ***n***(***r***), first appearing on a submicron scale but then annealing over a roughly 2 °C interval into patterns of elongated lines of low optical contrast (Fig. 1*B*) that are also oriented generally along ***n***(***r***). Some lines coarsen and extend along ***n***, while others form closed loops, 10 to 200 microns in extent, having a distinctive characteristic lens shape, elongated along ***n***(***r***) (Fig. 1 *C* and *D*). Sample textures from this evolution are shown in Fig. 1, with additional images in *SI Appendix*, Figs. S4–S9. Upon completion of the transition, apart from these loops, the texture is smooth and very similar to that of the N phase at high *T* (*SI Appendix*, Fig. S6). A small increase of *Δn* is observed at the transition (*SI Appendix*, Fig. S17), and *Δn* then increases continuously with decreasing temperature, *T*, behavior in agreement with that previously reported for the nematic order parameter (33).

**Fig. 2.**
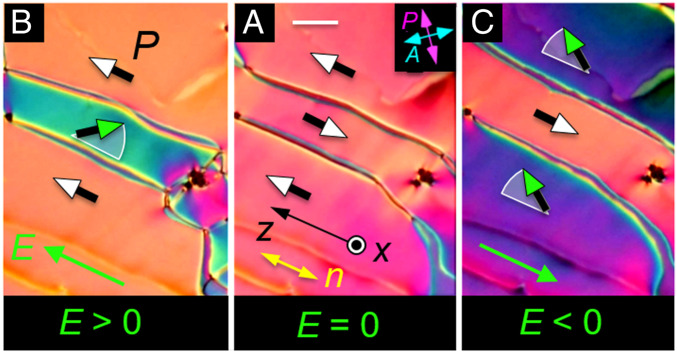
DTLM images showing polar Freedericksz twist transition in ferroelectric domains with opposite polar orientation at *T* = 120 °C. These domains, grown field-free upon cooling from the N phase to this temperature, have a polarization density ***P***. (*A*) Field-free initial state showing three domains separated by domain walls, each domain having ***n***(***r***) along the buffing direction ***z***. (*B*) Application of an ultra-small, positive test field *E*_z_ = 1 V/cm induces a birefringence color change resulting from in-plane reorientation of ***n***(***r***) in the center domain, leaving the upper and lower domains unchanged. (*C*) Application of *E*_z_ < 0 induces an in-plane reorientation of ***n***(***r***) in the upper and lower domains. There is little optical change or reorientation in the central domain. If the field is returned to *E = 0*, the system returns to the starting state (*A*). These observations demonstrate that the domains are polar and also enable the absolute determination of the direction of ***P***(***r***): domains that have the orientation preferred by the applied field do not reorient. In this experiment, ***P***(***r***) and ***n***(***r***) within the domains rotate about ***x*** but the field is not large enough to move the domain walls, which are pinned by the surfaces. The polarization vectors (shaded green) and circular arcs (white) depict the field-induced reorientation of ***P***(***r***) in the midplane of the cell: ***P***(***r***) does not reorient at the surfaces in this experiment, remaining parallel to the buffing direction. These field-induced reorientations with ***P***(***r***) starting nearly antiparallel to ***E*** are polar azimuthal Freedericksz transitions. The threshold field, *E*_P_ = (π/*t*)^2^(*K*_T_/*P*), estimated using the measured *P* ∼5 µC/cm^2^ at *T* =120 °C (Fig. 3), is *E*_P_ ∼1 V/cm, comparable to the fields employed here. *t* = 11 µm. (Scale bar, 30 µm.)

The N_F_ phase exhibits striking electro-optic behavior in DTLM, starting with the response to the application of tiny (<1 V/cm) in-plane electric fields. This sensitivity was exploited to probe and understand the static and dynamic changes of ***n***(***r***) and ***P***(***r***) in applied electric fields. Typical textures observed in these experiments, and the responses used to identify ferroelectric domains and determine their polarization orientation, are indicated in Fig. 2. In the absence of applied field, the LC director ***n*** in these cells is generally along the buffing direction ***z***, and we observe domains separated by distinct boundaries. As in Fig. 1, one set of domains responds only weakly to fields applied along ***z***, indicating that in these regions, ***P*** is already parallel to ***E*** and therefore that before the field is applied, ***P*** is everywhere either nearly parallel to or antiparallel to ***z***. In the domains where ***P*** is nearly antiparallel to ***E***, the polarization responds by rotating toward ***E***. The optical response to test fields makes the difference in polarity readily distinguishable, leaving no doubt that these domains are polar. The green vectors in Fig. 2 indicate the field-induced reorientation of ***P***(***r***) in the midplane of the cell. Tilting of the cell shows that this reorientation is azimuthal [𝜑(***r***)] about ***x***, with ***n***(***r***) remaining parallel to the (*y*, *z*) plane.

In an applied electric field, the ***n***(***r***) field of the preferred states becomes only slightly better aligned along the field direction, evidence that ***n*** and ***P*** are parallel. We considered the possibility that the N_F_ phase is biaxial, with ***n*** along ***z*** and secondary directors oriented preferentially parallel and normal to the cell surfaces. When such a biaxial phase grows in from a higher-temperature uniaxial phase, the cell generally exhibits arrays of characteristic disclination lines where 180° flips of the secondary directors have become trapped at one or both surfaces (39). If such structures were present in our cells, they would show up clearly in the polarized light microscope, but none are observed. We conclude that the bulk N_F_ condition is uniaxial, with ***n*** parallel to ***P*** in the absence of applied field. When an electric field is applied, ***n***(***r***) experiences electric torques through its coupling with ***P***, which we assume remains substantially locally parallel to ***n***, i.e., that the local optic axis is parallel to ***P***.

*SI Appendix*, Fig. S4, shows a larger area of the *t* = 11 µm cell in orientations having the average director along the crossed polarizer/analyzer direction (*SI Appendix*, Fig. S4 *A* and *F*–*H*) and at 45° to it (*SI Appendix*, Fig. S4 *B*–*E*), illustrating that the overall textural alignment gives reasonably good extinction between crossed polarizers but with some brighter regions allowed because of the softness of the anchoring as noted above. The images show polarization reversal driven by an adjustable DC in-plane electric field. The textures of ***n***(***r***) in the limiting states of plus or minus *E* are identically black but separated by a striking sequence of domain wall formation, coarsening, and disappearance, all in the weak DC field range −2 V/cm < *E* < 2 V/cm. The field-aligned states extinguish between crossed polarizers as in *SI Appendix*, Fig. S4*A*, meaning that that they have ***n***(***r***) everywhere parallel to z, and show a pink birefringence color in the third-order Michel-Levy band as in *SI Appendix*, Fig. S4*B*. The intermediate states have ***n***(***r***) in the (*y*, *z*) plane but with spatial variation of its azimuthal orientation 𝜑(***r***) about ***x***. This lowers the effective retardance of these regions, moving their birefringence down into the second- and first-order Michel–Levy bands and producing intense birefringence colors. The uniformly oriented domains obtained following field reversal are states in which the ***n***, ***P*** couple has been reoriented in the bulk LC and also flipped on the aligning surfaces, the latter mediated by domain wall motion.

The field-induced reorientations in the planar-aligned geometry of Figs. 1 and 2 are twist deformations of the azimuthal orientation of ***n***(***r***) about ***x***, having the form 𝜑(***r***) = 𝜑_c_(x) cos(π*x*/t) for small 𝜑_c_, where 𝜑_c_(*x*) is the reorientation pattern in the cell midplane. This deformation can be generated in a uniaxial dielectric N phase using an in-plane AC electric field to induce a twist Freedericksz transition, for which the threshold field will be given by *E*_D_ = (π/*t*)√(*K*/𝜀_o_Δ𝜀) (40). Assuming a cell gap *t* = 11 µm, typical nematic values of Frank elastic constant *K* ∼ 5 pN, and a dielectric anisotropy Δ𝜀 ∼ 5, one finds *E*_D_ ∼ 1,000 V/cm, giving an estimate which sets the field scale for typical in-plane dielectric nematic electro-optics. The fields required to produce the reorientations in the N_F_ phase in Figs. 1 and 2 are three orders of magnitude smaller. In small applied fields, electrical torque on the director field 𝝉_E_ = ***P*** × ***E*** comes from the coupling of field to polarization. With this polar coupling and ***P***(*x*) starting antiparallel to ***E***, our observed field-induced reorientations are polar Freedericksz transitions for which the torque balance equation is *K*_T_𝜑_xx_ + *PE*sin𝜑(*x*) = 0, where *K*_T_ is the twist elastic constant (41, 40). This gives a field threshold of *E*_P_ = (π/*t*)^2^(*K*_T_/*P*) and a cell midplane reorientation of 𝜑_c_(*E*) ≃ √[6(*E* − *E*_P_)/*E*_P_], so that a 𝜑_c_ = 0 to 𝜑_c_ = 90° reorientation occurs in the field range *E*_P_ < *E*_0–90_ < 1.4 *E*_P_. Measurements of *E*_0–90_ yield an experimental value of the threshold of *E*_P_ ∼ 1 V/cm, from which we can estimate *P*. Taking *K*_T_ to be in the range 2 pN < *K*_T_ < 5 pN gives a value for *P* in the range 3 µC/cm^2^ ≲ *P* ≲ 6 µC/cm^2^.

**Fig. 3.**
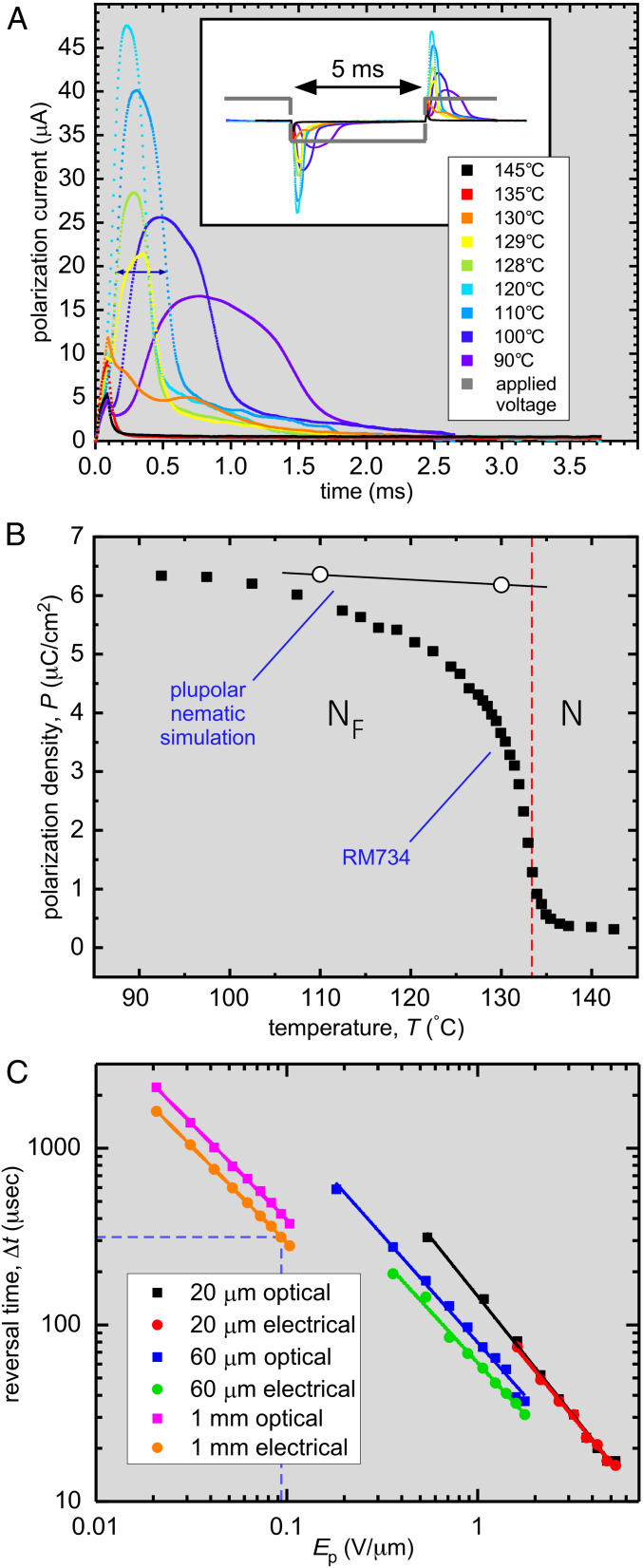
Characteristics of polarization reversal in an applied field. (*A*) Temperature dependence of the cell current with a 200-Hz square wave generating a field *E*_p_ = 95 V/mm applied in-plane to a *t* = 15-µm-thick cell with 1-cm-wide ITO electrodes spaced by *d* = 1 mm. In the I and N phases (*T* ≥ 133 °C), the current is small and capacitive. On cooling into the N_F_ phase, an additional current contribution appears, the area of which is independent of voltage and is equal to the net polarization reversal charge, *Q* = 2*PA*, where *A* = 15 µm × 1 cm is the effective cross-sectional area of the volume of LC material reoriented by the applied field. In the N_F_ phase, the polarization reversal current becomes a distinct peak that grows in area on cooling, indicative of an increasing polarization density, and the reorientation takes place more slowly, reflecting the increase of orientational viscosity. The double-headed arrow shows the reversal time at *T* = 110 °C (dashed drop lines in *C*). (*B*) The polarization density *P* of RM734 measured on cooling (black squares) saturates at *P* ∼6 µC/cm^2^ at the lowest temperatures. The open circles are values of *P* of the plupolar nematic calculated from the *POL* MD simulation of the N_F_ phase (Fig. 7 and *SI Appendix*, Sections S9 and S10). In the plupolar nematic, long-wavelength orientation fluctuations are suppressed, giving a *P* value determined by molecular-scale fluctuations and local packing. RM734 approaches the plupolar condition at low *T*. The region near the transition has not been studied in detail. (*C*) Field dependence of the reversal time 𝛥*t*, taken as the full width at half-height of the polarization or optical reversal pulse following a field step in a 100-Hz, bipolar, square-pulse train of peak amplitude *E*_p_ in planar-aligned cells with in-plane electrodes spaced by *d* = 20 µm, 60 µm, and 1 mm at *T* = 110 °C. The reversal time scales as *1/E*_p_ as expected for reorientation driven by ferroelectric torques. The dashed lines identify the measurement with *E*_p_ = 95 V/mm highlighted in *A*. The risetime 𝜏 = γ_1_/*PE* is ∼0.1𝛥*t*, giving a value of γ_1_ ∼0.1 Pa-s, comparable to the viscosity of 5CB at *T* =25 °C.

We also measured *P*(*T*) directly from the field-induced current associated with polarization reversal. We used both square- and triangle-wave driving fields in several different, two-terminal cell geometries, including an in-plane cell similar to that used for the electro-optics but with gold electrodes (*SI Appendix*, Fig. S3) 100-µm-thick sandwich cells with conventional gold electrodes and a 0.5-mm diameter, cylindrical capillary with the LC in a 150-µm gap between planar electrode faces oriented normal to the cylinder axis (*SI Appendix*, Fig. S3). Time integration of the current signals obtained using these geometries produced consistent values of the polarization density as a function of temperature. Typical polarization data obtained from the in-plane cell using a square-wave drive frequency f = 200 Hz are shown in Fig. 3. The cell current showed only small capacitive and resistive contributions in the N phase but, upon cooling into the N_F_ phase, became dominated by an additional peak which exhibited the characteristics of ferroelectric LC polarization current, carrying a driving amplitude-independent net charge *Q* (from which *P* was calculated) and exhibiting a risetime 𝜏 varying inversely as the drive amplitude (Fig. 3*C*). The resulting *P* increases continuously from small values at the transition, saturating at low *T* at a value *P* ∼ 6 µC/cm^2^, a value comparable to the ∼4 µC/cm^2^ found by Kikuchi et al. in the compound DIO (29). Polarization in the N–N_F_ transition temperature region may include pretransitional contributions in the N phase due to the divergence of 𝜀_‖_ (30), but this has not yet been studied in detail. The estimate above of *P* from the polar twist threshold *E*_P_ at *T* = 120 °C agrees well with the *P* data in Fig. 3, indicating that the magnitude of spontaneous polarization achieved field-free in domains grown and cooled from the N phase is comparable to that induced by a field in the N_F_ phase, as expected for a ferroelectric.

Further significance of *P* ∼ 6 µC/cm^2^ can be appreciated by calculating a polarization estimate *P*_e_ = *p/v*, where *p* = 11 Debye is the axial molecular dipole moment of RM734 (31) (*SI Appendix*, sections S9 and S10) and *v* is the volume/molecule in the phase, *v* = 325 cm^3^/mole = 540 Å^3^/molecule, assuming a LC mass density of 𝜌 = 1.3 g/cm^3^ (*SI Appendix*, sections S9 and 10). Using these parameter values and assuming complete polar ordering of the molecular long axes, we find *P*_e_ ∼ 6.8 µC/cm^2^, comparable to our measured *P* at low *T* and indicating that RM734 has extremely strong spontaneous macroscopic polar ordering, a condition impossible in any state with domains of competing polarization as in the proposed splay nematic, for example. This value of *P* is confirmed by our atomistic molecular dynamics (MD) simulations, which yield *P* ∼ 6.2 µC/cm^2^ in an equilibrated simulation of 384 molecules polar ordered in the N_F_ phase, as discussed below and in *SI Appendix*, sections S9 and S10. This *P* value is roughly six times larger than the highest polarization ever achieved in tilted calamitic or bent-core chiral smectic LCs (42, 43), is comparable to that found in polar columnar phases (44), and is well within the range exhibited by solid-state oxide (45) and organic (46) ferroelectrics. This result, combined with our textural observations, indicates that the N_F_ phase is a three-dimensional (3D), fluid, macroscopically homogeneous, polar uniaxial nematic phase. The agreement with the spontaneous *P* measured from the polar twist threshold indicates that this is the ferroelectric state.

**Fig. 4.**
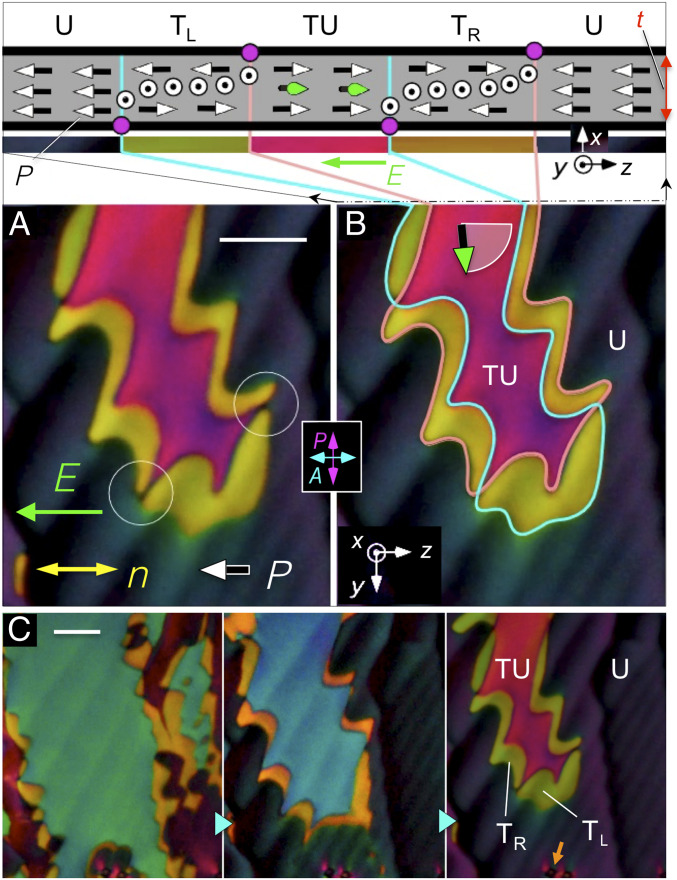
DTLM images of a large, twisted domain with surface polarization pointing to the right, surrounded by a uniform region with surface and bulk polarization pointing to the left, in the direction of an applied field. (*A*) Twisted domain (magenta) and structural elements ***P***, ***n***, and ***E***. (*B*) The section drawing shows the two-dimensional structure of the cell in the *x*, *z* plane along the top edge of the image: the uniform (U), field-preferred state of the background; the surface orientations reversing at the boundaries of the central domain; ***P*** in the twisted–untwisted (TU) state in the center of the domain, with the orientation in the middle of the cell indicated by green vectors, and the intermediate left- and right-handed twisted states T_L_ and T_R_ (olive and gold). π surface disclination lines (magenta dots) mediate polarization reorientation at the top (pink line) and bottom (cyan line) cell plates. Where the surface disclination lines overlap, the director is uniformly oriented along ***y*** through the thickness of the cell, giving extinction between the crossed polarizers (dark spots circled in *A*). In the absence of applied field, the left and right surface polarization states are energetically equivalent. (*C*) The central domain shrinks with increasing *E* field. The birefringence color changes from green to blue to pink as the rotation of ***P*** in the middle of the cell increases. *T* = 120 °C. *t* = 11 µm. Silica spheres (orange arrow) for visual size reference in the bottom of *C* have an apparent diameter of 4 µm (See *SI Appendix, Fig. S10*). (Scale bars, 35 µm.)

**Fig. 5.**
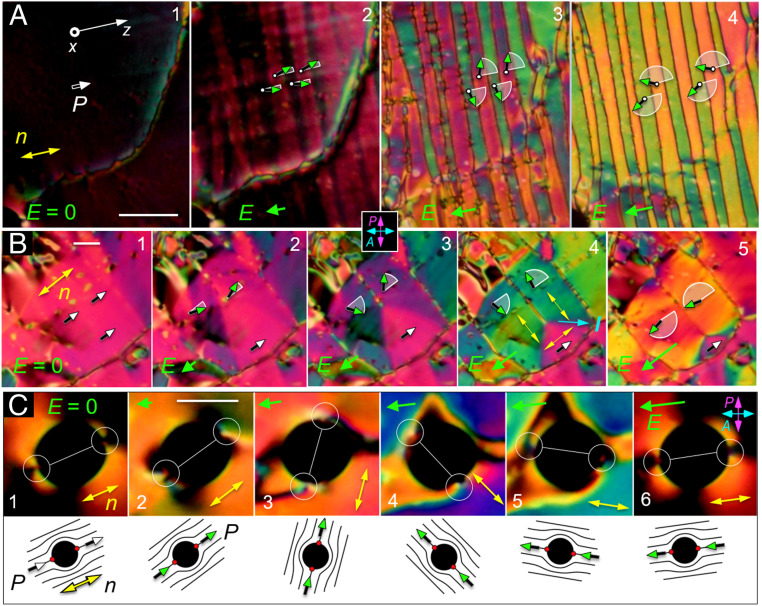
Common polarization reversal scenarios in RM734. Field-induced reorientation of ***P*** is indicated schematically using white arcs and green vectors. (*A*) Stripe formation. Applying a 5-Hz triangle-wave electric field with peak amplitudes in the range 0 < *E*_p_ < 10 V/cm to a region with an initially uniform in-plane director (panel 1) induces a periodic modulation in the orientation of ***n***(***r***) and ***P***(***r***) along ***z*** (a director bend wave; panel 2) whenever the field changes sign. As the applied field strength is increased (panels 3 and 4), the stripes form with sharper boundaries and have uniform internal orientation determined by the field strength. The zigzag arrangement of the director in successive stripes ensures that the normal component of ***P*** is constant across the stripe boundaries, so that there is no net polarization charge there. (*B*) Polygonal domains. During field reversal, polarization charge effects alternatively lead to the formation of tile-like domains with uniform ***n***(***r***). These polygons have sharp domain boundaries that are oriented such that ***P*⋅*l***, where ***l*** is along the boundary, is the same on both sides of the boundary, reducing space charge. The angular jump in ***n***(***r***) across the boundary highlighted in panel 4 is 90°. (*C*) Director field reorientation around inclusions. Air bubbles in the cell can be used to track the orientation of ***n***(***r***) in a reversing field. The director field near the bubble, sketched below each panel, is locally distorted, bending around the inclusion with splay deformations confined to two 180° wedge disclinations (red dots) located at opposite ends of the bubble. The blue color in panels 4 and 5 is indicative of a TU state of the kind shown in Fig. 4, with a surface disclination then moving out from the bubble boundary to give the final, uniform state seen in panel 6. (Scale bars, 40 µm in *A*, 30 µm in *B*, and 20 µm in *C*.)

Given these very large polarization values, we summarize here and detail in *SI Appendix*, section S2, several of the relevant features of high-polarization electro-optic, electrostatic, and elastic behaviors developed in the study of chiral smectic ferroelectric LCs, which can now be expected for the N_F_, some of which are reported here. These include the following: 1) One feature is polar twist Freedericksz transition (Fig. 1 and *SI Appendix*, Fig. S5). 2) A second feature is boundary penetration (47). The polar coupling to field limits the distance in which boundary- or defect-preferred orientations transition into bulk field-preferred orientations. This penetration length 𝜉_E_ = √*K*/*PE* ∼ 1 µm for *P* = 6 µC/cm^2^ and an applied field *E* = 1 V∕cm (Fig. 4). 3) A third feature is block polarization reorientation and expulsion of splay (splay-elastic stiffening) (48–55). A second self-penetration length, 𝜉_P_ = √𝜀*K*/*P*^2^ ∼ 0.1 nm, is the scale above which the polarization field can spontaneously expel spatial variation of orientation [∇**⋅*P***(***r***) = −𝜌_P_] that produces space charge, 𝜌_P_. The result is that low-energy elastic distortions of the ***n***, ***P*** couple are bend or twist only, with splay of ***n***(***r***) and ***P***(***r***) expelled from the bulk and confined to narrow reorientation walls, as in Fig. 5 *A* and *B* and *SI Appendix*, Fig. S4. The resulting polarization blocks can effectively screen applied field, producing, for example, the large threshold field required for the splay–bend Freedericksz transition in the N_F_ phase (*SI Appendix*, section S6). 4) A fourth feature is field-induced torques proportional to *E*. The balance of field-induced torque proportional to *PE* with viscous torques gives a characteristic reorientation risetime on the order of 𝜏 = γ_1_/*PE*, where γ_1_ is the nematic rotational viscosity (41, 56). The risetime 𝜏 = γ_1_/*PE* is ∼0.1𝛥*t*, where 𝛥*t* is the reversal time (Fig. 3*C*), giving a value of γ_1_ ∼ 0.1 Pa s, comparable to that of 5CB at *T* =25 °C. 5) The N–N_F_ phase transition is strongly affected by the polarization self-interaction, which suppresses the longitudinal modulation *δP*_z_ of ***P*** (50), producing the strong anisotropy of the polarization fluctuations in the N phase and rendering the transition mean-field (*SI Appendix*, section S3).

The electro-optic response of the N_F_ phase in a cell with an electric field applied in-plane shows uniquely polar features, with ***P***(***r***) reorienting in the (*y*, *z*) plane through an azimuthal angle 𝜑(***r***) about ***x*** determined by the local surface, elastic, and electric torques. Buffed surfaces stabilize two planar-aligned states (𝜑=0 and 𝜑=π) with opposite signs of ***P***(***r***), so the cell has four stable states, two that are uniform and two that are twisted, illustrated in Fig. 4. These equilibrium states are separated by π surface disclination walls (magenta dots in the section drawing of Fig. 4). If complete polarization reversal is to be achieved by an applied field, ***P*** must be switched on both surfaces. We refer to domain boundaries such as these, where both ***n*** and ***P*** reorient but maintain a fixed relative sign, as polarization-director (***P***-***n***) disclinations (***Pn***Ds). If the vectorial representation ***n***(***r***) is used to describe the nematic texture, then with only ***Pn***Ds the local orientation of ***P***(***r***) relative to ***n***(***r***) will be either parallel or antiparallel everywhere. In Fig. 4, an applied field preferring the uniform (U) dark state is deforming a central (magenta) domain that had polarization that was initially opposed to the field and is now partially rotated toward it (green vectors). This central domain forms a twisted–untwisted (TU) state in which the director twists along ***x*** from the surface-preferred alignment parallel to ***z*** at one cell plate through azimuthal angle 𝜑(*x*) to the field-aligned orientation in the midplane of the cell and then twists back to the surface-preferred alignment on the other glass plate. The field causes the central domain to shrink, moving and eventually eliminating the disclination walls in order to achieve complete polarization reversal. The motion of the walls on the two cell surfaces is different because the pinning strengths are different and spatially inhomogeneous, with the result that they do not remain in register, leading to the formation of the (olive) left- and (gold) right-handed twisted states T_L_ and T_R_ seen surrounding the central domain in Fig. 4 *A* and *B*. The color of the central TU domain (green, blue, or pink in Fig. 4*C*) varies with the degree of field-induced reorientation of ***n***, ***P*** in the sample midplane.

Several other modes of field-induced polarization reversal are shown in Fig. 5 and *SI Appendix*, Fig. S4. The initial response of a uniformly aligned region to an increasing in-plane DC field in the range 0 < *E* < 2 V/cm that opposes the local polarization is to form a zigzag modulation in the orientation of ***n***(***r***) and ***P***(***r***), illustrated in *SI Appendix*, Fig. S4, in which the nonzero spatial variation is 𝜕***n***(***r***)/𝜕*z*, along the director, making it a bend wave. Bend has a lower polarization space charge energy cost than a splay wave (which, with nonzero 𝜕***n***(***r***)/𝜕*x*, would generate stripes parallel to ***n*** rather than normal to it). As the field strength is increased, the degree of reorientation increases, and distinct boundaries appear between the half-periods of the modulation, separating stripes of uniform internal orientation. Fields of a few V/cm drive complete (+π, −π, +π, −π) reorientation of ***n*** in the cell midplane, at which point these boundaries become 2π walls suboptical in resolution, ∼𝜉_P_ in width. This process is even more dramatic with dynamic driving, as illustrated in Fig. 5*A*, which shows snapshots during field reversals generated by a 5-Hz AC triangle wave voltage with different amplitudes. As the field amplitude increases from 0 to *E*_p_ = 10 V/cm, the stripes become very regular and narrowly spaced. The herringbone pattern of polarization in the stripes gives an overall structure where *P*_z_ is constant, ensuring that there is no net polarization charge at the stripe boundaries, and where the backflow induced in each stripe matches that of its neighbor. The field is not strong enough to reverse the surfaces in this case. A different reorientation mode is illustrated in Fig. 5*B*, where field reversal leads to the formation of polygonal domains in which charge-stabilized areas of uniform ***P*** are bounded by sharp domain boundaries, each oriented along a vector ***l*** such that ***P*⋅*l*** has the same value on either side of the boundary, as shown in panel 4. As with the stripes above, this geometry reduces the net polarization charge on the line. Similar structures are found in high-*P* chiral smectic ferroelectrics (57) and in ferromagnets (58). The textures of the charge-stabilized domains can also be employed to visualize directly the reorientation of ***P***(***r***) under applied field, as shown in Fig. 5*C*, where a circular air bubble enables tracking of the polar orientation of ***n***(***r***) during field reversal. The director is anchored tangent to the bubble surface, resulting in a director field that is largely bent around the bubble, with splay concentrated in two 180° wedge disclinations (red dots). ***n***(***r***) in the area surrounding the bubble is parallel to the line connecting these defects. The ***n***(***r***), ***P***(***r***) structures observed with increasing reversal field strength are sketched below each panel.

### Nematic Ferroelectrohydrodynamics.

**Fig. 6.**
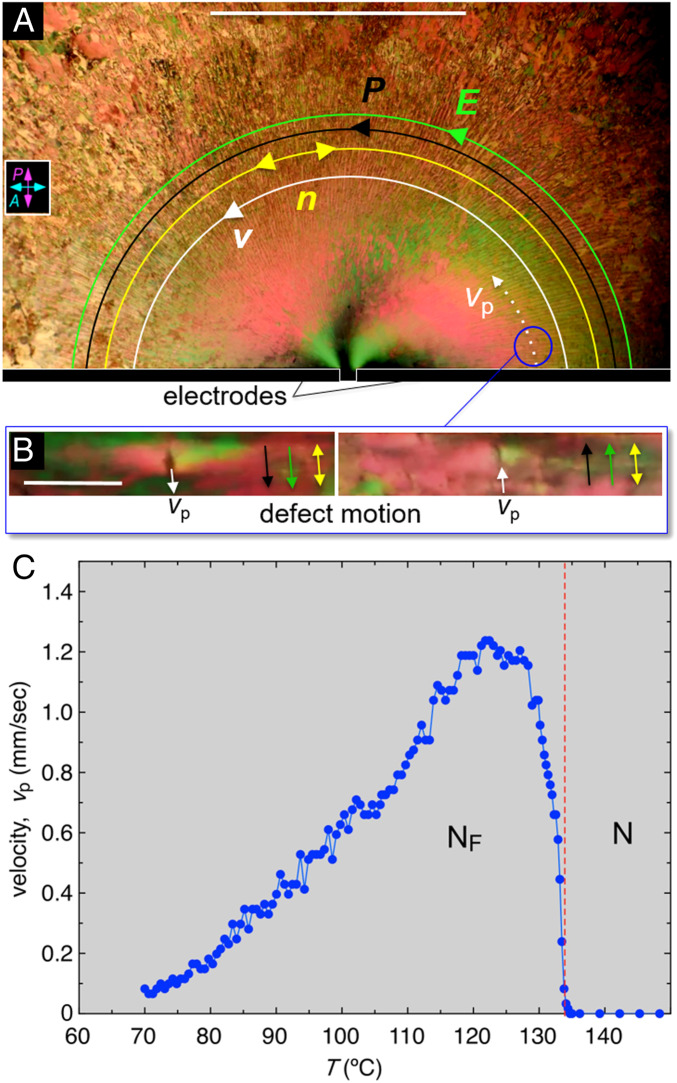
Field-induced flow in the ferroelectric nematic phase. (*A*) DTLM image of a *t* = 10-µm-thick, planar-aligned cell of RM734 between untreated glass plates, in the N_F_ phase at *T* = 120 °C. The black bars at the bottom are two evaporated gold electrodes on one of the plates, separated by a *d* = 60 µm gap. The electrodes are outlined in white for clarity. Only the upper edges of the electrodes and the adjacent part of the cell are shown. A square-wave voltage with *V*_p_ = 3 V, 0.1 Hz, is applied to the electrodes, producing an electric field in the plane of the cell. This field drives a pattern of defect motion and fluid flow over the entire field of view, with the defect velocity ***v***(***r***) (white arrows) parallel to the applied field, ***E***(***r***) (green), which is tangent to half-circles centered on the electrode gap (*SI Appendix*, Fig. S12). Where the defects are dense, their motion transports the surrounding fluid. When the field is applied, the entire region shown here moves along the field lines. This image, captured during field reversal, shows a periodic array of bend domain walls normal to the director (yellow) and the applied field, as in Fig. 5*A*, in this case along radial lines. (*B*) Typical defect in the texture moving along the applied field direction (down in *Left* and up in *Right*), in the location circled in *A*. (*C*) Temperature dependence of the magnitude of the initial defect velocity along the white dashed track in *A* following a field reversal. There is no flow in the N phase but on cooling into the N_F_ phase, the velocity increases rapidly with increasing *P* before decreasing again at lower *T* because of the increasing viscosity. A similar dependence on *T* is observed whether heating or cooling. (Scale bars, 1 mm in *A* and 100 µm in *B*.)

The polarization density of the N_F_ phase creates a fluid which is extraordinarily responsive both to external applied fields and to internally generated polarization space charge. While the discussion above has focused on the effects of field-induced molecular reorientation, the most interesting and useful effects of the N_F_ may be its ferroelectrohydrodynamic or ferroelectrorheological behavior, exemplified by the observations shown in Fig. 6 and *SI Appendix*, section S7. In this experiment, RM734 is filled into a *t* = 10 µm cell with random-planar alignment of ***n***. An in-plane electric field is applied using a pair of gold electrodes evaporated with a *d* = 60 µm gap onto one glass plate, visible at the bottom of Fig. 6*A*. A square wave voltage with *V*_p_ = 5 V applied between the electrodes generates an electric field distribution where ***E***(***r***) is uniform in the electrode gap and in the surrounding area is directed along half-circular arcs centered on the gap (*SI Appendix*, Fig. S15 *A*, *Inset*). In the N_F_ phase, this field induces flow of localized defects (Fig. 6*B*) and their surrounding fluid with a velocity field ***v***(***r***,*t*) locally parallel to ***E***(***r***) and changing direction with the field, suggesting an electric body force density ***F***(***r***) = 𝜌(***r***)***E***(***r***), where 𝜌(***r***) is a positive electric charge density. When ***E***(***r***) goes through *E* = 0 during field reversal, flow ceases, and the director field breaks up into ***P***-reversal bend-domain bands like those shown in Fig. 5*A* as it rotates alternately through +π and −π, giving the radial texture seen in Fig. 6*A*. Thus, dynamically ***P***(***r,****t*) is everywhere parallel to ***E***(***r,****t*) and ***v***(***r,****t*) when voltage is applied. The fact that the product ***P***(***r***)**⋅*E***(***r***) is unchanged by applied field reversal and yet ***v***(***r***) changes sign indicates that 𝜌(***r***) does not change sign with ***P***(***r***), i.e., that the driving has caused the fluid to become charged. The structure of polarization reversal bands in the neighborhood of the electrode gap is shown in detail in *SI Appendix*, Fig. S16.

We measured *v*_p_, the initial value of the defect velocity upon field reversal at the location indicated in Fig. 6*A*. This velocity depends dramatically on temperature, as shown in Fig. 6*C*, with flow being essentially absent in the N phase and commencing upon cooling through the N–N_F_ transition. The velocity eventually decreases with decreasing *T*, presumably because of the increasing viscosity of the LC.

The experiments show that applied electric field promotes the creation of regions with positive charge density. Charging of the N_F_ by AC applied fields is to be expected due to the bulk polarity of the phase. Electrode surfaces contact N_F_ material where the direction of *P* alternates in time. The N_F_, because of its polar symmetry, has diode-like, polarity-dependent resistance that can also depend on the sign and nature of the charge carrier. The bulk charge mobility along *z* in the N_F_ phase may also depend on field direction. Beyond this, there will be a variety of charging effects due to the linear coupling of *P* and flow. Let us consider, for example, steady, incompressible nematic laminar flow; then the director is generally nearly parallel to the velocity, and ***v***(***r***) = *v*(***r***)***n***(***r***). Since ∇**⋅*v***(***r***) = 0 we have ∇**⋅*n***(*s*) = [ln *v*(*s*)]/𝜕*s*, where *s* is the position variable along the flow: where the velocity increases, the director splays inward. However, in the N_F_ phase we have ***P***(***r***) = *P****n***(***r***), where *P* is the constant polarization magnitude, so that laminar flow produces polarization charge density 𝜌_*P*_(*s*) = −*P*∇**⋅*n***(*s*) = −*P*𝜕[ln *v*(*s*)]/𝜕s, the sign of which depends on whether *P* is aligned along *v* or opposed to it. Complex flows will thus produce complex patterns of polarization charge. Reorientation of ***P*** generates displacement current, ***J*** = 𝜕***P***/𝜕*t*, which is locally normal to ***P***(***r***) and, if driven by electric field, gives a highly anisotropic contribution to the net electrical conductivity, σ_⟂_ = *P*^2^/γ_1_ for ***E*** ⟂ ***P***, and σ_||_ = 0 for ***E*** || ***P*** (43). For RM734, we obtain σ_⟂_ ∼10^−3^/Ωcm, which is in the semiconducting range. Under these circumstances, accumulation of one sign of charge in the fluid can occur when an applied AC field gets out of phase with polarization reversal. Additional inherent asymmetries, such as differences in mobility or chemical character between positive and negative ionic impurities, or an intrinsic tendency for splay distortion of the ***P***(***r***) field itself, can also contribute.

## Atomistic MD Simulation

We carried out MD simulations directed toward gaining an understanding of how features of molecular architecture, interactions, and correlations are related to the polar ordering of the N_F_ phase. These calculations used a simulation box containing 384 RM734 molecules (*SI Appendix*, Fig. S18) with periodic boundary conditions, equilibrated in the *NPT* ensemble at *P* = 1 atm for a range of temperatures spanning the N and N_F_ phases, using the APPLE&P force field (59) successfully applied in previous studies of nematic (60) and twist-bend (61) phases. More details of the simulations can be found in *SI Appendix*, sections S9 and S10.

**Fig. 7.**
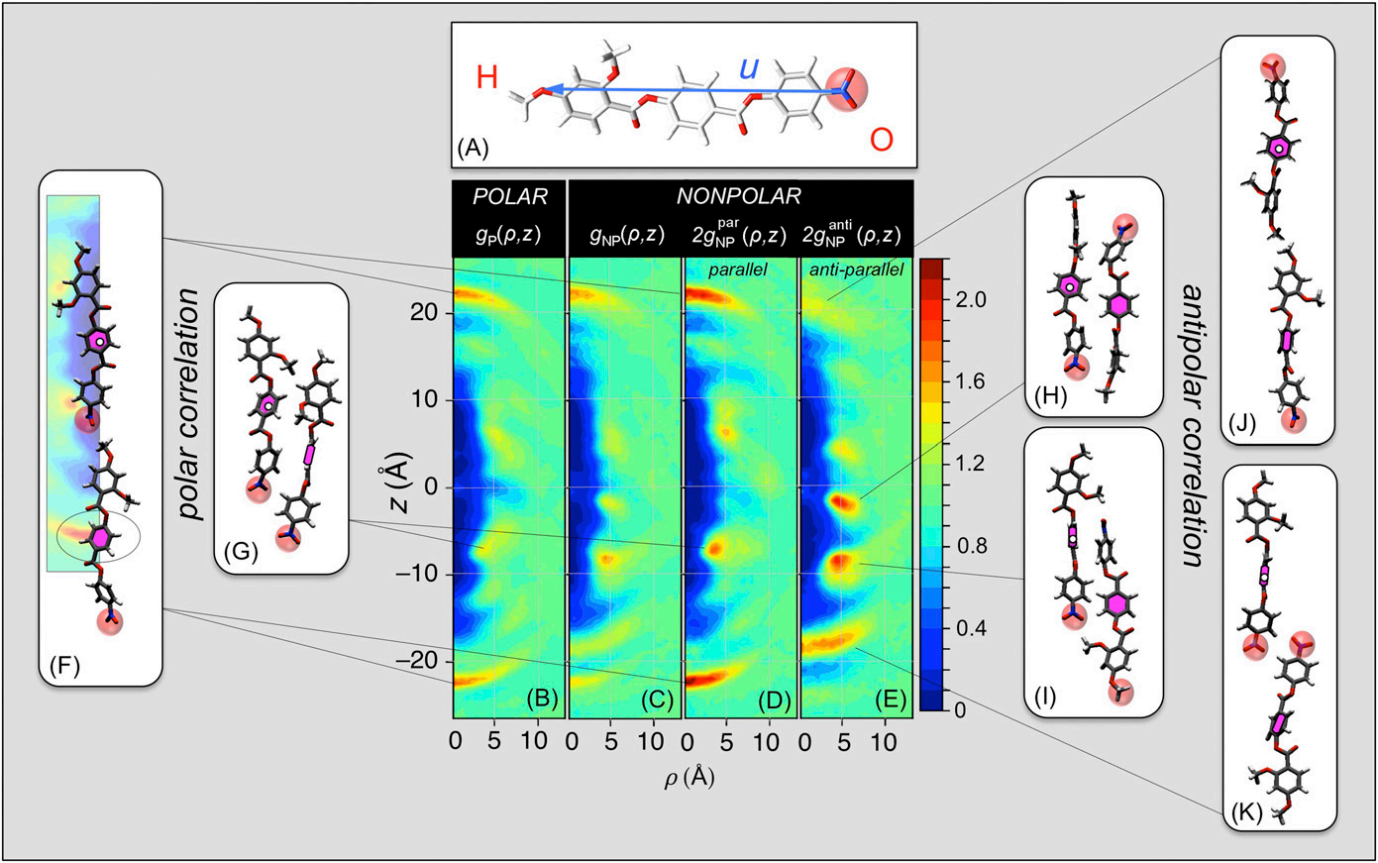
Results of atomistic molecular dynamic simulations probing molecular-scale organization leading to polar order. (*A*) RM734, showing its geometrical long axis vector ***u***, terminating at the nitro- (*O*) and methoxy (*H*) molecular ends. A nanoscale volume containing 384 molecules is equilibrated into two distinct LC states: a *POLAR* system with all polar molecular long axes, u, along +z and a *NONPOLAR* system with half along +z and half along −z. Equilibration of the molecular conformation and packing is readily achieved, but end-to-end flips are rare, so the simulated states remain in the polar or nonpolar limit of equilibrated nematic order. (*B*–*E*) Molecular positional/orientational pair correlation functions: conditional probabilities of molecular centers (magenta), about centers fixed at the origin (white dots). (*B*, *F*, and *G*) The *POL* simulation shows directly the dominant pair correlations adopted by molecules that are polar ordered, in the form of conditional probability densities, *g*(ρ,*z*), of molecular centers (magenta fill) around a molecule with its center (white dots) at the origin and long axis ***u*** along *z*. The *g*(ρ,*z*) are 𝜑-averaged to be uniaxially symmetric, reflecting the uniaxial symmetry of the N and N_F_ phases. They exhibit a molecule-shaped, low-density region [*g*(ρ,*z*) ∼0] around the origin resulting from the steric overlap exclusion of the molecules; an asymptotic constant value at large ρ giving the normalized average density [*g*(ρ,*z*) = 1]; and distinct peaks indicating preferred modes of molecular packing. This analysis reveals two principal preferred packing modes in the *POL* system: (*B* and *F*) polar head-to-tail association stabilized by the attraction of the terminal nitro and methoxy groups and (*B* and *G*) polar side-by-side association governed by group charges along the molecule, nitro-lateral methoxy attraction, and steric interactions of the lateral methoxys. (*D* and *E*) The *NONPOL* system exhibits distinct correlation functions for antiparallel and parallel molecular pairs, *g*_NP_^anti^(ρ,*z*) and *g*_NP_^par^(ρ,*z*). (*E*, *H*, and *I*) The preferred antiparallel packing gives strong side-by-side correlations, governed by group charges along the molecule, and (*E*, *J*, and *K*) weaker antipolar nitro–nitro end-to-end association. (*D*, *F*, and *G*) The parallel correlations in the *NONPOL* system are the most relevant to the stability of polar order in the N_F_ phase as they are determined by the inherent tendency of the molecular interactions for polar ordering in the presence of enforced polar disorder. Comparison of *B* and *D* shows identical preferred modes of parallel association in the two systems, with the *POL* system correlations being even stronger in the *NONPOL* system. This is clear evidence that the polar packing motifs giving the correlation functions (*B*) and (*D*), exemplified by the sample *POL* MD configurations (*F*) and (*G*), stabilize the polar order of the ferroelectric nematic phase.

The simulations probe the equilibration of RM734 in two distinct condensed LC states: 1) polar (*POL*), a polar nematic state generated by equilibrating a starting condition that is perfectly ordered in the +***z*** direction of the polar molecular long-axis vectors ***u***, which point from the nitro (O) to the methyl (H) end of each molecule as seen in Fig. 7*A*, and 2) nonpolar (*NONPOL*), a weakly polar, nematic state generated by equilibrating a starting condition with no net polar order (50%/50% division of the ***u*** vectors along +*z*/−*z*). Each molecule can then be labeled as OH or HO, depending on its orientation (whether ***u*** is along +*z* or −*z*, respectively). Equilibration with respect to the internal molecular arrangements of these two systems is readily achieved through orientational and internal molecular fluctuations as well as diffusive molecular motion. Their equilibrated states are distinct, however: the steric packing of the anisotropic molecules makes head-to-tail molecular flipping events extremely rare during the simulation space–time volumes, so the equilibrations obtained have an effective constraint of no molecular flipping.

These *POL* and *NONPOL* states represent the extremes of equilibrated polar order and polar disorder in the simulation volume. Limiting the simulations to these states, i.e., not considering molecular flips in a simulation of polar order, may seem like a significant shortcoming. However, when we use the *POL* state to calculate *P*, we obtain polarization densities that match those of RM734 at low *T* (Fig. 3*B*), implying that at low temperatures the ordering of RM734 becomes that of the simulated *POL* state, making this an ideal model system for exploration of the molecular correlations leading to polar order.

The positional pair-correlation functions, *g*_P_(ρ, *z*) and *g*_NP_(ρ, *z*), of the equilibrated *POL* and *NONPOL* systems are shown in Fig. 7 *B*–*E*, where for the *NONPOL* system, *g*_NP_(ρ, *z*) = *g*_NP_^par^(ρ, *z*) + *g*_NP_^anti^(ρ, *z*) is the sum of the correlations between the molecular pairs with relative parallel or antiparallel orientations of ***u***. The *g*(ρ, *z*) are 𝜑-averaged conditional probability densities of molecular centers around a molecule with its center at the origin and long axis along *z* and thus are uniaxially symmetric in (ρ, 𝜑, *z*) cylindrical coordinates, reflecting the uniaxial symmetry of the N and N_F_ phases. They all exhibit a molecule-shaped, low-density region [*g*(ρ, *z*) ∼ 0] around the origin resulting from the steric overlap exclusion of the molecules, an asymptotic constant density at large ρ, and distinct peaks indicating preferred modes of molecular packing. The normalized average density is <*g*(ρ,*z*)> = 1. If we were to consider a similarly equilibrated system of rods marked O and H on their ends but which were otherwise symmetric (e.g., hard spherocylinders marked as either OH or HO) then both of the orientational states would have identical pair-correlation functions, *g*_P_(ρ, *z*) = *g*_NP_(ρ, *z*) = 2*g*_NP_^par^(ρ, *z*) = *2g*_NP_^anti^(ρ, *z*). The *g*(ρ, *z*) of RM734, in contrast, show a number of striking differences that directly exhibit the effects of its structural and electrostatic polarity on the packing of neighboring molecules.

The *POL g*_P_(ρ, *z*) in Fig. 7*B* shows the equilibrated, local molecular packing preference in the limit of polar order, i.e., in the system with the maximum number of contacts between like-oriented molecules. Its prominent features are sharp, on-axis arcs at (ρ = 0, *z* = ±22 Å), indicating on-average coaxial molecular association into polar chain-like (OH–OH–OH) associations having a center-to-center spacing along *z* of the molecular length, 22 Å, stabilized by the electrostatic attraction of the nitro and methoxy ends of the molecules (Fig. 7*F*), and off-axis peaks at ρ = 5 Å, *z* = ±6 Å, indicating polar side-by-side association (Fig. 7*G*). These correlations indicate that specific electrostatic interactions between oppositely charged groups on the two molecules (e.g., between positively charged terminal or lateral methoxy H atoms and negatively charged nitro O atoms) play a dominant role in stabilizing such pair configurations.

The *NONPOL* system enforces the maximum number of molecular contacts between molecules of opposite orientation. In this situation of maximum polar disorder, possible equilibrated molecular correlations could range from being 1) dominantly antiparallel end-to-end [e.g., OH–HO–OH chains, with side-to-side polar correlations, as in the bilayer smectics of strongly polar molecules (62)] to being 2) polar end-to-end (a mixture of OH–OH–OH and HO–HO–HO chains with the OH–HO interactions side by side). RM734 is distinctly in the latter category as, remarkably, the principal polar ordering motifs of Fig. 7 *F* and *G* are even stronger in the *NONPOL* system than in the *POL* (compare Fig. 7 *B* and *D*), and the antipolar correlations are largely side by side. The OH–HO end-to-end antipolar association depicted in Fig. 7*J* is present but weak, as is the HO–OH end-to-end pairing of Fig. 7*K*. The latter is dominant in the crystal phase (32) but not as a mode of achieving antipolar ordering in the *NONPOL* system. It appears from these results that the polar correlations identified in the *POL* system, and persisting in the *NONPOL* system in the maximal presence of enforced polar disorder, must be those responsible for stabilizing the N_F_ phase.

The *POL* simulation equilibrates a state in which end-to-end flipping is kinetically arrested and the periodic boundary conditions suppress long-wavelength orientation fluctuations (λ_x_ > 55 Å and λ_z_ > 70 Å). The remnant short-ranged fluctuations lead to pair correlations which, as we have shown in Fig. 7, are confined to the volume ρ <10 Å and *z* < 30 Å about the origin, molecular neighbor separation scales which are well within the dimensions of the simulation box. These conditions create a “plupolar” [*plus quam* polar (63)] equilibrium state of constrained polar ordering yielding the simulated *P* values shown in Fig. 3 (open circles). Comparing these polarization values with the RM734 data shows 1) that in the plupolar state, the fluctuations that lead to the phase transition are clearly suppressed, while the remnant short-range fluctuations give a *P* value exhibiting only a weak dependence on temperature, and 2) that this *P* gives a good account of the polarization density of the N_F_ at low temperature, evidence that at low *T* the N_F_ phase approaches a comparable plupolar-like condition where there are only short-range fluctuations and where the simulated *g*(ρ, *z*) faithfully represent their correlations.

## Materials and Methods

### Synthesis of RM734.

4-[(4-nitrophenoxy)carbonyl]phenyl 2,4-dimethoxybenzoate (RM734; Fig. 1*A*) is a rod-shaped mesogen first synthesized by Mandle et al. (31). It was reported to have an isotropic (I) phase and two additional phases with nematic character, with transition temperatures as follows: I–187 °C–N–133 °C–N_X_–X. Our preparation is based on general synthetic reactions and uses procedures only slightly modified from those described in the literature cited (*SI Appendix*, section S1).

### Observations of Response to Applied Electric Field.

Experimental cells were made by filling LC samples between glass plates coated with lithographically patterned ITO or gold electrodes and spaced to a desired gap, *t*. Both transparent capacitor and in-plane electrode geometries were employed. Experiments were performed in temperature-controlled environments, with electro-optic observations carried out using DTLM with cells mounted on the rotary stage of a research microscope and imaged in transmitted light between polarizers. The sign and magnitude of the in-plane birefringence were determined using a Berek compensator (*SI Appendix*, Fig. S17). Polarization measurements were made by using transimpedance electronics to integrate the current in response to an applied electric field, using several in-plane geometries and a glass capillary cell with coaxial electrodes (*SI Appendix*, section S1).

### Data Availability.

Data, simulations, and videos are publicly available at DOI: 10.17605/osf.io/ZKFTW.

## Supplementary Material

Supplementary File

Supplementary File

Supplementary File

Supplementary File

Supplementary File

Supplementary File
