# Supplementary material for: First-principles experimental demonstration of ferroelectricity in a thermotropic nematic liquid crystal: Polar domains and striking electro-optics
Source: Proc Natl Acad Sci U S A. 2020 Jun 10;117(25):14021–31. doi: 10.1073/pnas.2002290117 (PMC7322023; doi:10.1073/pnas.2002290117)
Supplement: Supplementary File [file pnas.2002290117.sapp.pdf]

PNAS
www.pnas.org

SUPPLEMENTARY INFORMATION

First-Principles Experimental Demonstration of Ferroelectricity in a Thermotropic Nematic Liquid Crystal: Polar Domains and Striking Electro-Optics

Xi Chen[1], Eva Korblova[2], Dengpan Dong[3], Xiaoyu Wei[3], Renfan Shao[1], Leo Radzihovsky[1], Matthew A. Glaser[1], Joseph E. Maclennan[1], Dmitry Bedrov[3], David M. Walba[2], Noel A. Clark[1]

[1]*Department of Physics and Soft Materials Research Center, University of Colorado, Boulder, CO 80309, USA*

[2]*Department of Chemistry and Soft Materials Research Center, University of Colorado, Boulder, CO 80309, USA*

[3]*Department of Materials Science and Engineering, University of Utah, Salt Lake City, UT 84112, USA and Soft Materials Research Center, University of Colorado, Boulder, CO 80309, USA*

Corresponding Author - Noel Clark (*noel.clark@colorado.edu*)

Abstract

We report the experimental determination of the structure and response to applied electric field of the lower-temperature nematic phase of the calamitic literature compound 4-[(4-nitrophenoxy)carbonyl]phenyl2,4-dimethoxybenzoate (RM734). We exploit its electro-optics to visualize the appearance, in the absence of applied field, of a permanent electric polarization density, manifested as a spontaneously broken symmetry in distinct domains of opposite polar orientation. Polarization reversal is mediated by field-induced domain wall movement, making this phase ferroelectric, a 3D uniaxial nematic having a spontaneous, reorientable, polarization locally parallel to the director. This polarization density saturates at a low temperature value of ~ 6 $\mu C/cm^2$, the largest ever measured for a fluid or glassy material. This polarization is comparable to that of solid state ferroelectrics and is close to the average value obtained by assuming perfect, polar alignment of molecular long axes in the nematic. We find a host of spectacular optical and hydrodynamic effects driven by ultra-low applied field (E~1 V/cm), produced by the coupling of the large polarization to nematic birefringence and flow. Electrostatic self-interaction of the polarization charge renders the transition from the nematic phase mean-field-like and weakly first-order, and controls the director field structure of the ferroelectric phase. Atomistic molecular dynamics simulation reveals short-range polar molecular interactions that favor ferroelectric ordering, including a tendency for head-to-tail association into polar, chain-like assemblies having polar lateral correlations. These results indicate a significant potential for transformative new nematic science and technology based on the enhanced understanding, development, and exploitation of molecular electrostatic interaction.

www.pnas.org/cgi/doi/10.1073/pnas.2002290117

TABLE OF CONTENTS

Section S1 – *MATERIALS AND METHODS* ***(Figs. S1-S3)***........3

Section S2 – *PLANAR–ALIGNED LARGER-AREA CELL TEXTURE* ***(Fig. S4)***........6

Section S3 – *THE N–N_F AND N_F–N PHASE TRANSITIONS* ***(Figs. S5-S10)***........8

Section S4 – *COMBINING PPR AND PND DOMAIN WALLS* ***(Fig. S11)***........17

Section S5 – *SPLAY IN THE N AND N_F TEXTURES* ***(Fig. S12)***........18

Section S6 – *SANDWICH CELL EO BEHAVIOR & THE FREEDERICKSZ TRANSITION* ***(Figs. S13,S14)***........19

Section S7 – *CHARACTERISTICS OF FIELD-INDUCED FLOW* ***(Figs. S15,S16)***........22

Section S8 – *VARIATION OF UNIAXIAL BIREFRINGENCE WITH TEMPERATURE* ***(Fig. S17)***........24

Section S9 – *ATOMISTIC SIMULATION METHODOLOGY* ***(Fig. S18)***........25

Section S10 – *ATOMISTIC SIMULATION RESULTS* ***(Figs. S19-S22)***........25

Section S11 – *MOVIE CAPTIONS* **(Movies 1 - 5)**........32

SUPPLEMENTARY INFORMATION REFERENCES........34

Section S1 *– Materials and Methods*

Materials – *RM734 - 4-[(4-nitrophenoxy)carbonyl]phenyl 2,4-dimethoxybenzoate* (***Fig. S1***) is a rod-shaped molecule about 20 Å long and 5 Å in diameter, with a longitudinal electric dipole moment of about 11 Debye. First reported by Mandle et al. [1,2], it was found to melt at T = 140ºC and have an isotropic (I) phase and two additional phases with nematic-like character, with transition temperatures on cooling as follows: I – 188ºC – N – 133ºC – N_X. Our synthetic scheme, shown in ***Fig. S1***, is based on general synthetic reactions and procedures only slightly modified from those described in the literature cited. The synthesized compound has very similar phase transition temperatures to those reported by Mandle (***Fig. S2***).

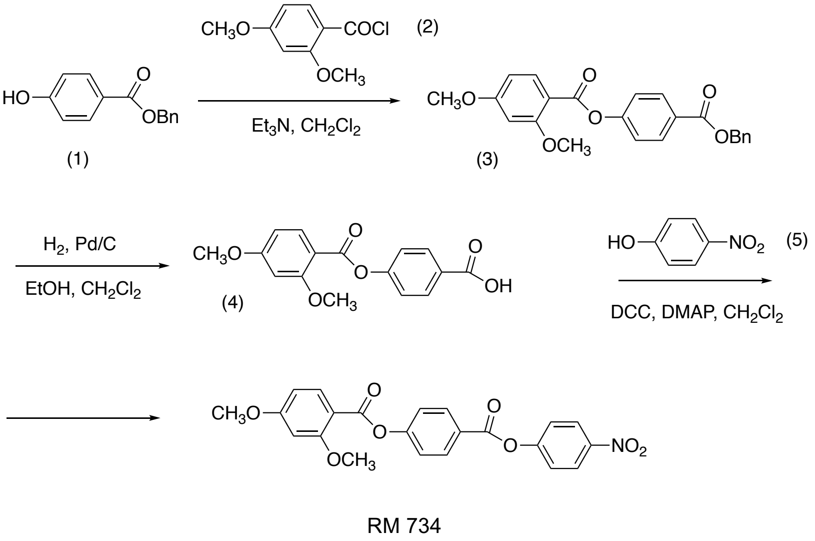

Figure S1: Synthesis scheme for RM734.

Reagents, solvents, and starting materials were used as purchased from qualified suppliers without additional purification. Reactions were performed in oven-dried glassware under an atmosphere of dry argon. Purification by flash chromatography was performed with silica gel (40–63 microns) purchased from Zeochem AG. Analytical thin-layer chromatography (TLC) was performed on silica gel 60 F_{254} TLC plates from Millipore Sigma (Darmstadt, Germany) Compounds were visualized with shortwavelength ultraviolet (UV). Nuclear magnetic resonance (NMR) spectra were obtained using a Bruker Avance-III 300 spectrometer. NMR chemical shifts were referenced to $CHCl_3$ (7.24 ppm for 1H, 77.16 ppm for ^{13}C).

4-[(benzyloxy)carbonyl]phenyl 2,4-dimethoxybenzoate (3) – Benzyl 4-hydroxybenzoate (**1**) (5.69 g, 25 mmol) and 2,4-dimethoxybenzoyl chloride (**2**) (5.00 g, 25 mmol) were dissolved in CH_2Cl_2 (200 mL), and cooled to 0°C. Triethylamine (3.03 g, 30 mmol, 4.2 mL) was then added dropwise. The reaction mixture was stirred overnight at T = 25ºC, then poured into a saturated aqueous solution of NH_4Cl (200 mL) and extracted with dichloromethane. The combined organic layers were washed with water, brine, dried over $MgSO_4$, filtered and concentrated at reduced pressure. The crude product was purified by flash chromatography (silica gel, CH_2Cl_2/2% EtOH), to afford ester **2** as a white solid (9.4 g 96%, Mp. 89-92°C).

4-(2,4-dimethoxybenzoyloxy)benzoic acid (4) – A solution of the benzyl-protected carboxylic acid (**3**) (9.4g, 24 mmol) in CH_2Cl_2 (100 mL) and EtOH (100 mL) was first evacuated and purged with argon, then 10% Pd/C catalyst (2 g) was added. The argon atmosphere was replaced by hydrogen gas, and the reaction mixture was stirred at room temperature for two hours. Hydrogen was pumped out of the system and the flask was purged thoroughly with argon. The mixture was filtered through Celite and the solvents were removed under reduced pressure. This gave product as a white powder (6.99 g 96%).

4-[(4-nitrophenoxy)carbonyl]phenyl 2,4-dimethoxybenzoate – To a suspension of compound **4** (4.38 g, 15 mmol) and 4-nitro phenol (**5**) (2.02 g, 15 mmol) in CH_2Cl_2 (125 mL) was added DCC (6.00 g, 30 mmol) and trace of DMAP. The reaction mixture was stirred at room temperature for three days, then filtered, washed with water, 5% CH_3COOH, water, and brine, then dried over $MgSO_4$, filtered, and concentrated at

reduced pressure. The resulting product was purified by flash chromatography (silica gel, CH_2Cl_2/2% EtOAc) followed by crystallization from CH_3CN. The product is a white crystalline solid (3.92 g 62%). 1H NMR (300 MHz, $CDCl_3$) ∂ ppm: 8.36 -8.25 (m, 4H), 8.10 (d, 1H) 7.46 -7.37 (m, 4H), 6.60-6.55 (m, 2H), 3.94 (s, 3H), 3.91 (s, 3H). ^{13}C NMR (75 MHz, $CDCl_3$) ∂ ppm: 165.51, 163.73, 162.90, 162.64, 156.11, 155.82, 145.56, 134.74, 131.99, 125.69, 125.40, 122.76, 122.62, 110.53, 105.13, 99.16, 56.18, 55.75.

Differential scanning calorimetry measurements of RM734 are shown in ***Fig. S2***.

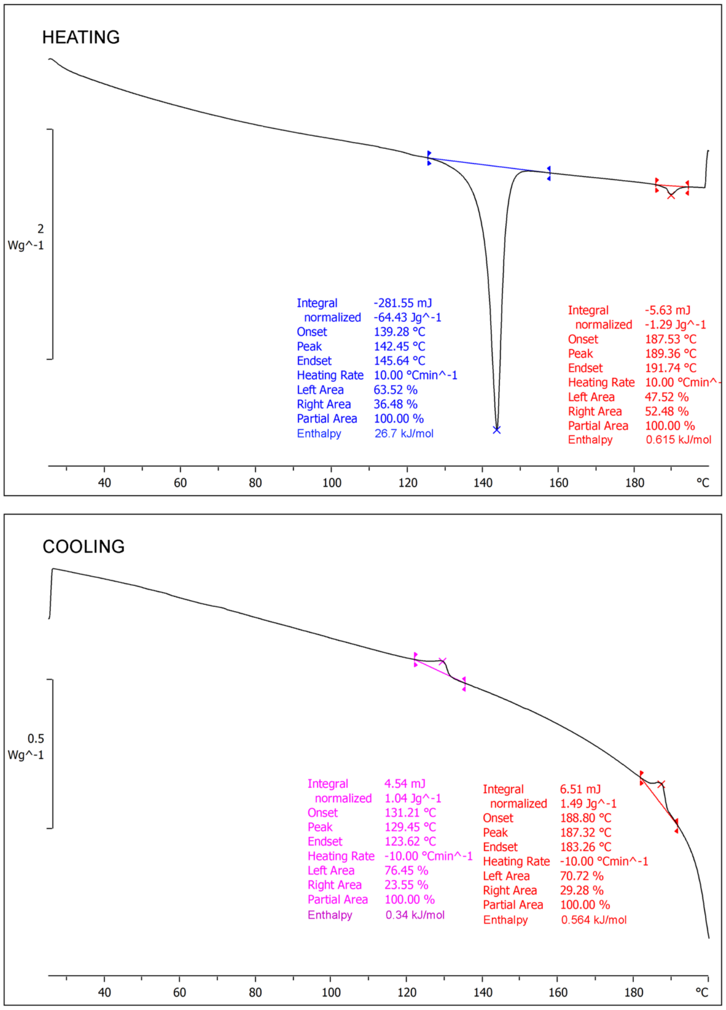

Figure S2: DSC measurements of RM734. The heating and cooling rates were respectively ±10°C/min. The heats of transition were computed using a molecular weight of 423.38.

Methods – Electrooptic Measurement – The liquid crystal phase sequence and phase transition temperatures were determined by polarized light microscopy using a Nikon Eclipse E400 POL microscope equipped with an Instec STC200 temperature-controlled hot stage. DSC was performed using a Mettler DSC823e differential scanning calorimeter. Electrooptic measurements were made using a Zeiss microscope, a Spectra Physics 105-1 HeNe laser, an EZ Digital FG-8002 function generator, and a Tektronix TDS 2014B oscilloscope.

Polarization Measurement – Polarization was measured by time integration of the current through two-terminal LC cells in which saturated polarization reversal was achieved. In-plane field cells, such as that in ***Fig. S3A***, and the cylindrical, coaxial end-to-end electrode glass capillary cell in ***Fig. S3B*** were used.

Polarization and electrooptic measurements were carried out, in addition, in a 15 μm-thick Instec cell with two, 1 cm-long rectangular ITO electrodes patterned a distance 1 mm apart on one substrate, a geometry similar to that of the in-plane switching cell shown in ***Fig. S3A***. The surfaces of the cell were treated with a planar alignment layer rubbed 87^0 from the electrode edges, the buffing direction ***z*** thus making an angle of 3^0 with the in-plane electric field in the gap between the electrodes.

Texture Cells - Unless otherwise noted, the optical textures and domain structures of RM734 reported in the paper were observed in a t = 11 μm-thick LC Vision cell with a pair of 5 mm-long ITO strip electrodes spaced by d = 1.04 mm on one surface, giving an in-plane electric field. The plates had bidirectionally buffed polymer alignment normal to the electrode edges forming the gap, which gave weak anchoring alignment of the director in the direction of the applied field (***Fig. S4***).

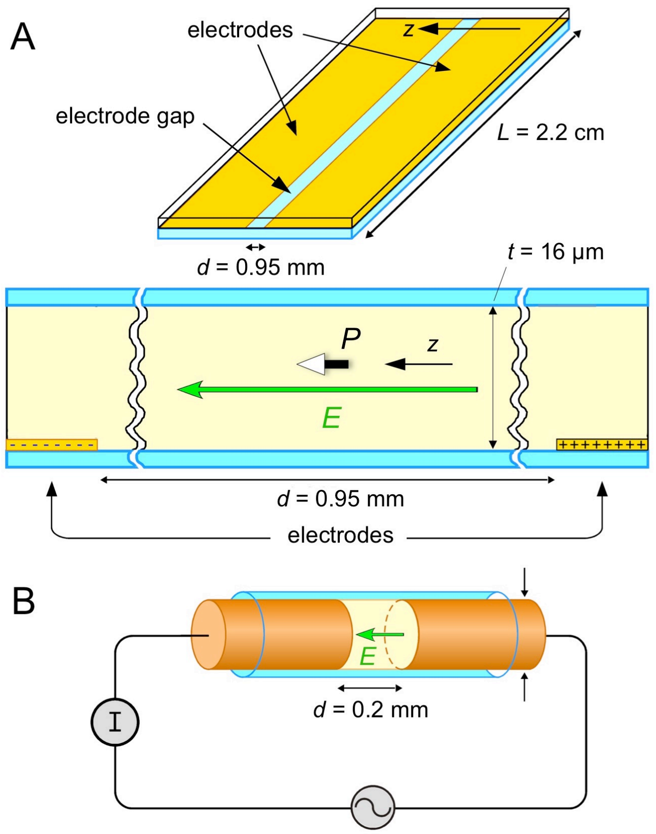

Figure S3: Polarization measurement cells. (A) In-plane switching cell. This cell was made from a glass slide coated with a pair of gold strip electrodes 2.2 cm long and d = 0.95 mm apart, with a top glass cover, and spacers giving a 16 μm LC thickness. The cell surfaces were thoroughly cleaned with acetone and methanol before assembly and there were no alignment layers. The cell was t = 16 μm thick and L = 2.2 cm long (the vertical dimensions are greatly exaggerated in the Figure). Since the cell thickness is much smaller than the gap between the electrodes, a voltage applied between the two electrodes produces a uniform alignment of the ferroelectric polarization P between the electrodes. (B) Capillary geometry. Two copper wires 0.55 mm in diameter with flat ends were inserted into a cylindrical glass capillary of slightly larger inner diameter that was previously filled with liquid crystal. The gap between the ends of the electrodes was 0.2 mm.

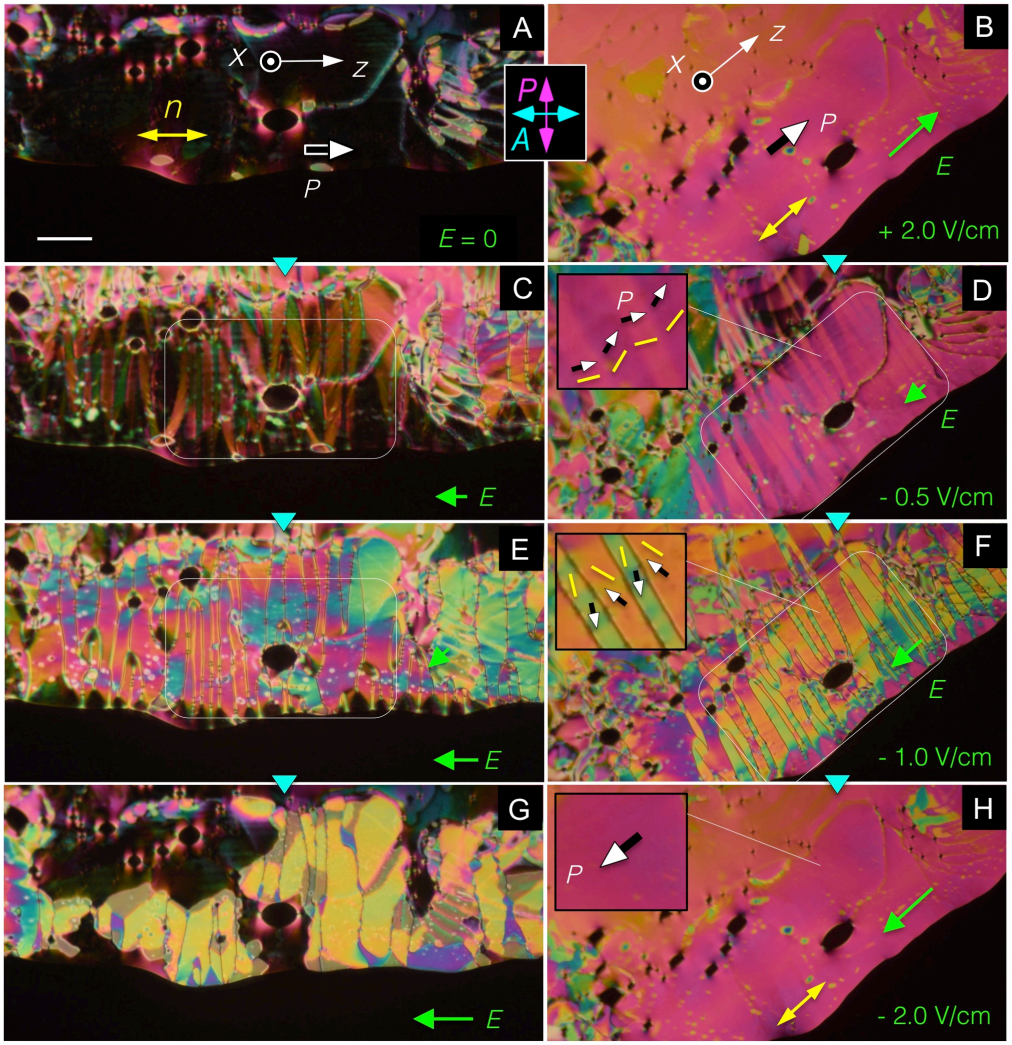

Figure S4: A larger area of the t = 11 μm test cell seen in ***Figures 1*** and **2** observed in DTLM, in orientations having the average director either along the polarizer/analyzer direction (**A, F–H**) or at 45º (**B–E**). The lower black area and the black ellipses are bubbles. (**A**) In the absence of applied field, the director $\boldsymbol{n}(\boldsymbol{r})$ is generally aligned along the rubbing direction $\boldsymbol{z}$, producing good extinction. Experiments with small probe fields applied along the rubbing direction confirm that the average $\boldsymbol{P}(\boldsymbol{r})$ is directed toward the right. Vertical white lines show the electrode edges. (**B**) Rotating the cell reveals the pink birefringence color of the aligned domains (in the third-order Michel-Levy band). An applied field, E = +2 V/cm parallel to $\boldsymbol{P}$, stabilizes the zero-field orientation. (**C,D**) Reversing the field to E ~ -0.5 V/cm begins the reorientation process, with bands in the texture corresponding to alternating rotation directions of the $\boldsymbol{n},\boldsymbol{P}$ couple. (**E,F**) At E ~ -1 V/cm the $\boldsymbol{n},\boldsymbol{P}$ couple has rotated by $\delta\varphi$ ~ ±90º, forming regions of different signs of rotation separated by boundary walls. These regions are birefringent rather than extinguishing because $\boldsymbol{n}(\boldsymbol{r})$ twists non-uniformly across the thickness of the cell. (**G,H**) At E ~ -2 V/cm, the rotations of the $\boldsymbol{n},\boldsymbol{P}$ couple are nearly +π or -π, with the regions of opposite rotation now separated by narrow 2π walls. This is immediately followed by a process in which domain boundaries nucleate holes whose growth leads to the elimination of the domain walls and the formation of large regions of uniform $\boldsymbol{n}$ and $\boldsymbol{P}$, a state that is identical to that of (**A, B**) but with $\boldsymbol{P}$ reversed. This polarization reversal scenario takes place for fields $|E|$ < 2 V/cm and is repeatable. Scale bar = 70 μm.

***Section S2** – High Polarization Electro-Optical, Electrostatic, and Elastic Effects*

Director field response **–** Given the very large polarization values of the N_F phase, it is useful to summarize several of the relevant features of polar electro-optic, electrostatic, and elastic behavior, developed in the study of chiral smectic ferroelectric LCs, which can now be expected for the N_F: (***i***) *Polar twist Freedericksz transition* [3,4] - We take the uniform equilibrium state to have ***P*** along ***z***, with ***n***(***r***) and ***P***(***r***) parallel to the (*y,z*) plane. In small applied fields, electrical torque on the director field $\boldsymbol{\tau}_E = \boldsymbol{P} \times \boldsymbol{E}$ comes from the coupling of field to polarization. Applying this coupling in the description of the twist Freedericksz transition, the equation describing the azimuthal orientation field $\varphi(x)$ across the thickness of an otherwise uniform cell becomes $K_T\varphi_{xx} + PE\sin\varphi = 0$, with the threshold characteristics given above. (***ii***) *Boundary penetration* - Solving $K\varphi(z)_{zz} + PE\sin\varphi(z) = 0$ instead in the *y,z* plane, with the boundary condition $\varphi(z{=}0) = 0$, using the one-elastic constant (*K*) approximation, and applying an electric field to stabilize $\varphi(z) = 180^\circ$ at large z, a π reorientation wall is established in the LC near $z = 0$, given by $\varphi(z) = 4\tan^{-1}[1 - \exp(-z/\xi_E)]$. The ferroelectric field penetration length $\xi_E = \sqrt{K/PE}$ gives the approximate width of the wall [5], the distance that the effects of local orientational pinning such as the wall can penetrate into the neighboring LC, assuming the latter has ***P*** held in place by ***E***. The penetration depth is $\xi_F \sim 1\ \mu$m for an applied field $E = 1$ V/cm).

(***iii***) *Block polarization reorientation and expulsion of splay (splay-elastic stiffening)* [6-13] - Spatial variation of ***P***(***r***) generally results in bulk and surface polarization charge density, given respectively by $\rho_P = \nabla\cdot\boldsymbol{P}(\boldsymbol{r})$ and $\sigma_P = \boldsymbol{P}_s\cdot\boldsymbol{s}$. The electric field generated by the bulk charge opposes the bulk distortion of ***P***(***r***) that caused it, producing a bulk energy $U_P = ½\int dv\ \nabla\cdot\boldsymbol{P}(\boldsymbol{r})\nabla\cdot\boldsymbol{P}(\boldsymbol{r}')[1/|(\boldsymbol{r} - \boldsymbol{r}')|]$. Assuming a periodic transverse modulation $\delta\boldsymbol{P}_y(\boldsymbol{r})$ of amplitude $P\delta\boldsymbol{n}_y$ and wavevector q_y, so that $\nabla\cdot\boldsymbol{P}(\boldsymbol{r}) = \partial P_y(y)y = iq_yP_z\delta n_y$ in our geometry, we have an elastic energy density $U_{sp} = ½[K_sq_y^2 + 4\pi P^2/\varepsilon]\ |\delta n_y|^2$, meaning that the polarization term will be dominant for $q_y < \pi\sqrt{2}/\xi_P$, where $\xi_P = \sqrt{\varepsilon K/P^2}$ is the polarization self-penetration length. Since for $P = 6\ \mu$C/cm^2 we have $\xi_P \sim 0.1$ nm, this dominance will act down to molecular length scales. The result is that low-energy elastic distortions of the ***n,P*** couple allow only bend, with splay of ***n***(***r***) and ***P***(***r***) expelled from the bulk and confined to reorientation walls of characteristic width ξ_P. On the other hand, if we consider a longitudinal modulation $\delta P_z(y,z)$, the additional electrostatic free energy density will be $U_P = ½[4\pi P^2q_z^2/\varepsilon(q_z^2 + q_y^2)]\ |\delta P_z|^2$ [8].

(***iv***) *Field-step reorientational response* - The dynamics of polarization reorientation and the electro-optic (EO) response to changes in applied field are complex, depending on elastic, viscous, surface, and flow-induced torques in addition to that of the field. However, with the application of a large field step the electrical torques initially dominate and these determine the risetime of the optical response. The balance of field and viscous torques gives a characteristic reorientation risetime on the order of $\tau = \gamma_1/PE$, where γ_1 is the nematic rotational viscosity [4,14] (***Fig. 3C***). The risetime $\tau = \gamma_1/PE$ is $\sim 0.1\Delta t$, where Δt is the reversal time (***Fig. 3C***), giving a value of $\gamma_1 \sim 0.1$ Pa sec, comparable to that of 5CB at $T = 25^\circ$C.

(***v***) *The N–N_F phase transition* is strongly affected by the polarization self-interaction, which suppresses the longitudinal modulation δP_z of ***P*** [7], resulting in considerable anisotropy of the polarization fluctuations in the N phase, and rendering the transition mean-field, discussed in ***Sec. S3*** below.

Charge screening – All such polarization-based effects are reduced by free space charge, such as ionic impurities in the LC and its containing surfaces [9,10], ionization of the LC itself, and charge injected from the electrodes, all of which tend to screen the polarization charge. In SmC* FLC cells, when the polarization is small ($P < 20$ nC/cm^2) the bound polarization charge can be substantially screened but for large

polarizations (P > 100 nC/cm^2) the free charge supply can be exhausted and polarization effects manifested. For the largest SmC* polarizations (P ~ 800 nC/cm^2), the polarization charge is largely unscreened and the polarization effects are quite dramatic [15]. Thus, the SmC* FLC literature provides examples of screened and unscreened FLC behavior from which we can infer that the polarization charge of the N_F phase is largely unscreened by ions. The DC conductivity of our RM734 sample is σ ~ 10^{-7} (Ohm-cm)$^{-1}$, which is small enough to contribute little to the polarization measurement current (***Fig. 3A***) and not to affect the N-phase Freedericksz transition, but large compared to typical 5CB for example, which is σ ~ 10^{-10} (Ohm-cm)$^{-1}$. Estimating the Debye length λ_D using the ion density from σ gives λ_D ~ 1mm, consistent with the observation that applied in-plane fields appear to be generally uniform and unscreened over millimeter dimension areas as in ***Fig. S4***, so the Debye length must be this size or larger.

SECTION S3 – THE N–N_F AND N_F–N PHASE TRANSITIONS

Ferroelectric domains – As is evident in ***Figs. 4,S5-S11***, the optical texture of the N phase in our planar-aligned cells is locally smooth. This texture roughens upon cooling toward the N_F phase, developing a random pattern of micron-size domains extended along ***n***, which, once in the N_F phase, coarsen into a smooth texture very similar to that of the nematic but marked by a pattern of smooth lines, some of which extend for long distances generally parallel to ***n*** and others which form closed loops to make macroscopic, lens-shaped domains, as seen in ***Figs. 1,S6***. The experiments presented in ***Figs. 1,S5,S11*** show that these lines demarcate areas of opposite polarization density, all formed in the absence of applied electric field. This remarkable evolution and the response of these domains to applied field are at the heart of the phenomenon of ferroelectricity, giving rise to its characteristic behaviors of macroscopic polar ordering and hysteretic dielectric behavior. In the weakly buffed cells studied here and in unbuffed cells, for thicknesses in the range 3 μm < t < 15 μm, the domain formation is similar to that reported here. Domain walls are generally extended along ***n*** and may have some characteristic spacing early in the coarsening process (***Fig. S9***), but toward the end of coarsening the spacing and loop size become very irregular - they appear to get pinned to the surfaces. The weak buffing in the t = 11 μm cells used here did not exhibit an in-plane polar alignment preference.

Generally, the domain walls are visible because they have a width comparable to the cell thickness, and they appear to have an intra-wall director with a boundary condition that tends to keep ***n*** locally parallel to the wall (***Fig. 6A***). A resulting feature of these walls, evident in ***Figs. 1,S5,S7,S11***, is that wherever they run exactly parallel to ***n***(***r***), they lose optical contrast and can no longer be seen in DTLM. This means that at these places there is no reorientation of ***n***(***r***) upon crossing the domain boundary: ***n*** is in the same direction inside and outside of the lens-shaped domains and through the wall, whereas ***P*** changes sign. We refer to these as pure polarization reversal (PPR) lines. PPR lines show that the differing states of the low-temperature phase found across domain walls differ only in the sign of ***P***, and that P is therefore a suitable order parameter for describing the transition, *i.e.*, that the N_F phase is a proper ferroelectric. PPR surfaces form upon cooling through the N–NF transition as the correlated volumes having predominately a particular sign of ***P***(***r***) grow in size and the polarization fluctuations become slow and glassy. The resulting three-dimensional domains ultimately establish an equilibrium degree of order, encoded in the magnitude of P, and confine the transitions to the opposite sign of P in neighboring regions into PPR surfaces of a local structure that includes an internal nematic sheet, the central surface of the wall where P actually changes sign. In thin cells, these PPR walls have the appearance of lines but they are of course three-dimensional, with a structure controlled in part by the aligning surfaces of the cell. In a bulk 3D system, the PPR walls must form some sort of closed-shell structures.

With this observation, we now have two distinct kinds of defect lines in our cells where $\boldsymbol{P}(\boldsymbol{r})$ can change sign relative to the lab frame, **z**: the PPR lines in which $\boldsymbol{P}(\boldsymbol{r})$ flips by 180° but $\boldsymbol{n}(\boldsymbol{r})$ is unchanged, and the π–disclination (***Pn***D) lines of ***Figs. 2*** and ***S5***, in which $\boldsymbol{P}(\boldsymbol{r})$ and $\boldsymbol{n}(\boldsymbol{r})$ rotate together, maintaining a fixed relative orientation. Observations of the PPR lines show that they avoid making angles ψ relative to $\boldsymbol{z}$ larger that about 45°. This is seen, for example, in the lens domains where the PPR line is forced to be a closed loop. The line, rather than forming a continuously circular domain wall, where ψ would at two locations get to be 90°, instead jumps from $\psi \sim -45°$ to $\psi \sim +45°$ in order to complete the loop.

Similar behavior is also shown in ***Fig. S7*** for an extended line, where such a jump is maintained until ψ can be become sufficiently small without it. π-PPR and π-***Pn***D lines can combine to make 2π walls in the uniquely ferroelectric way shown in ***Figs. S5, S11***.

Modeling the N-N$_F$ phase transition – The low-temperature phase of RM734 has been proposed to be a "splay nematic" phase [16,17,18], a splay-bend ground state of the sort initially described theoretically by Hinshaw et al. [19], in which the packing frustration of electrically and structurally polar (pear-shaped) molecules is relieved by the spontaneous adoption of local splay deformation, with 3D space filled by planar (x,z) sheets of splay ($\partial n_y/\partial y$) alternating in sign of splay and therefore also in sign of polarization (see Ref. [18], Fig. 11). However, we find that: (***i***) the N_F develops a large, macroscopic polarization as it grows in field-free through the transition from the N phase, and (***ii***) the ultimate polarized state at low temperature is effectively saturated, exhibiting no other significant birefringence or internal structural change upon cooling in the N_F phase and with little response to large applied fields tending to increase $\boldsymbol{P}$ (i.e., $\boldsymbol{E}$ parallel to $\boldsymbol{P}$). These conditions appear to preclude any significant cancellation of P by opposed splay domains.

We propose that the ground state of the N_F phase has, aside from fluctuations, uniform $\boldsymbol{P}$ and that it is a proper ferroelectric phase, where P is the principal order parameter and phase stability originates from a combination of short-range interactions that favor neighboring molecular long axes to be parallel and with the same polar orientation (***Fig. 1***), and long-range dipole-dipole interactions. Under this condition, the N–N_F transition would be viewed as Ising-like, with the N phase being a collection of molecules with long axes on average parallel to $\boldsymbol{n}$ but with disordered polar directions ($\langle p_i \rangle = 0$), and the N_F phase also having long axes on average parallel to $\boldsymbol{n}$ but with polar order $N\langle p_i \rangle = P$. Ignoring for the moment the dipole-dipole interactions, with only ferroelectric short-range interactions this transition would be predicted to be second-order with 3D critical exponents of the Ising universality class [20].

Mertelj et al. have observed paraelectric pretransitional effects, including the softening of the splay elastic constant, K_s, and a diverging dielectric anisotropy $\Delta\varepsilon$ in the N phase, that indicate growing polarization correlations, and flexoelectric coupling between these fluctuations and director splay as the transition is approached from above, observations which they have interpreted using a Landau-de Gennes model [17,18,21]. The free energy of Ref [18], $f = ½ [\tau(T)(1 + \xi(T)^2q^2)\delta P_z(q)^2 + K_s q_y^2\delta n_y(q_y)^2] + \gamma q_y \delta n_y(q_y) \delta P_z(q_y)$, where $\xi(T)^2 = b/\tau(T)$, $\tau(T) \propto (T\text{-}T_c)/T_c$, and $\boldsymbol{q} = \boldsymbol{q}_z + \boldsymbol{q}_y$, includes Ornstein-Zernicke polarization fluctuations about $q = 0$ (no splay modulation), originating from short-range polar interactions, and the flexoelectric coupling of P_z to director splay. This model successfully describes a mean field-like behavior of $K_s(\tau)$ and $\Delta\varepsilon(\tau)$. This free energy is consistent with the N_F phase having uniform $\boldsymbol{P}$, with a weak tendency for splay that is suppressed by the polarization charge effects discussed above, if the energy cost of the defects required to introduce splay modulation is included.

An additional pretransitional effect not yet adequately described is the striking anisotropy of the pretransitional and coarsening correlations in the fluctuations of $\boldsymbol{P}$ through the transition (***Fig. S8, S9***), with the domains becoming increasingly extended along $\boldsymbol{z}$, until they end up as polar regions separated by PPR

walls running largely parallel to $\boldsymbol{z}$. Given the significant anisotropies introduced into the N_F phase by space charge effects, it is natural to consider that the polarization charge energy U_P associated with electric dipole-dipole interactions may also affect the phase transition and pretransition fluctuations. The resulting interaction energy combines short-ranged ferroelectric and long-ranged dipole-dipole forces.

The critical behavior of such systems has been studied extensively in an effort to understand certain magnetic materials that have short-range ferromagnetic exchange forces, but where the long-range dipolar interactions are also important [22,23,24]. In these systems, short-ranged interactions are included in a model Hamiltonian as nearest-neighbor Ising or Heisenberg-like, and the long-ranged interactions are calculated explicitly. Renormalization group analysis shows that the long-range interactions make the magnetic correlations dipolar-anisotropic near the transition in the high temperature phase [25,26], extending them along $\boldsymbol{z}$ by strongly suppressing longitudinal charge density ($\partial P_z/\partial z$) fluctuations [22,23]. Specifically, starting with the free energy expression Eq. (1) from ref. [18] and adding the dipole-dipole interaction term U_P from above, the structure factor for fluctuations in P_z becomes $\langle P_z(\boldsymbol{q})P_z(\boldsymbol{q})^* \rangle = k_B T\chi(\boldsymbol{q})$ where $\chi(\boldsymbol{q}) = [\tau(T)(1 + \xi(T)^2q^2) + (2\pi/\varepsilon)(q_z/q)^2]^{-1}$. The dipole-dipole (third) term produces extended correlations that grow as $\xi(\tau)$ along x and y but as $\xi(\tau)^2$ along z [23], suppressing $\chi(\boldsymbol{q})$ for finite q_z as is observed qualitatively from the image sequences of the textures upon passing through the phase transition, and from their optical Fourier transforms in ***Fig. S9***. Because of this anisotropy, the correlation volume in this model grows in 3D as $V \sim (\tau)^4$ rather that the isotropic $V \sim \xi(\tau)^3$, reducing the upper marginal dimensionality of the transition to 3D, making the transition mean-field-like with logarithmic corrections, rather than fluctuation-dominated with 3D Ising universality [27].

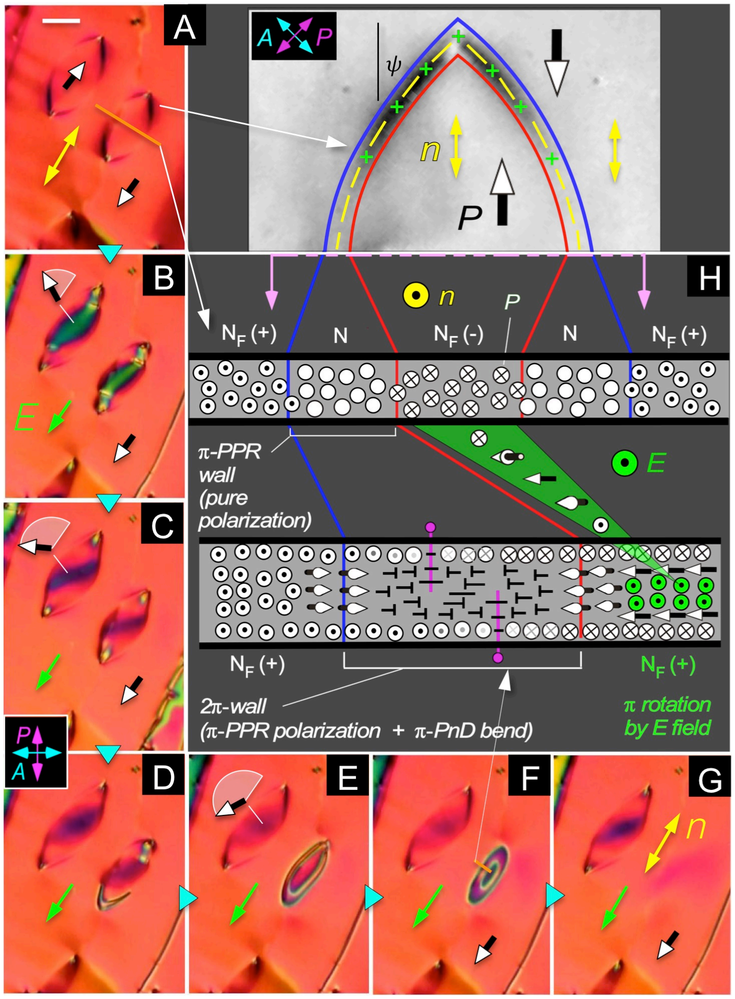

Figure S5: Step by step through the life of a lens-shaped N_F domain in an applied reversal field increasing in the range $0 < E < 1$ V/mm. (**A**) Virgin domains before the field is applied. Yellow arrows show the director ***n***, green the applied field ***E***, and black the polarization, ***P***. The director at the domain wall tends to follow the wall, otherwise ***n*** is uniform within each domain. The domain wall is invisible at the widest part of the lens, indicating that the wall does not involve reorientation of ***n***, only reversal of the polarization – a pure polarization reversal (PPR) wall. The lenticular shape of the domain is due the interaction of ***n*** inside the wall with the surface buffing, making PPR lines stable only for ψ <~ 45º. (**B**) In a small applied field (E ~ 0.1 V/mm), the domain interior reorients through an azimuthal angle φ ~ 90º. The area outside the domain, does not reorient, indicating that it is already aligned with the field. (**C**) Reorientation approaches 180º. (**D–F**) A reorientational disclination loop nucleates in the cell mid-plane to accommodate the induced 180º reorientation of ***n***, and moves in response to the applied field to surround the second domain. (**F**,**G**) The domain shrinks as the disclination loop collapses. The first lenticular domain also responds to the field but maintains its shape, its boundary apparently trapped more strongly. (**H**) Cross-sectional sketches of the domain structure before and during field application. The initial domain boundary is a PPR polarization wall with no disclinations in ***n***. In the region between N_F(+) and N_F(-) , the system must pass through $\boldsymbol{P} = 0$, *i.e.*, be nematic. The field-induced reorientation inside the disclination loop (green) adds an additional 180º rotation (a polarization-director disclination, ***Pn***D) to the domain wall, as well as surface twist disclinations inside the loop which connect the bulk field-induced reorientation to the surfaces. The green swoop indicates the field-induced reorientation of the domain interior, causing the formation of a compound π-PPR / π-***Pn***D boundary to make a 2π wall. The reorientation on the surface is mediated by π twist disclinations (magenta). Scale bar = 30 μm.

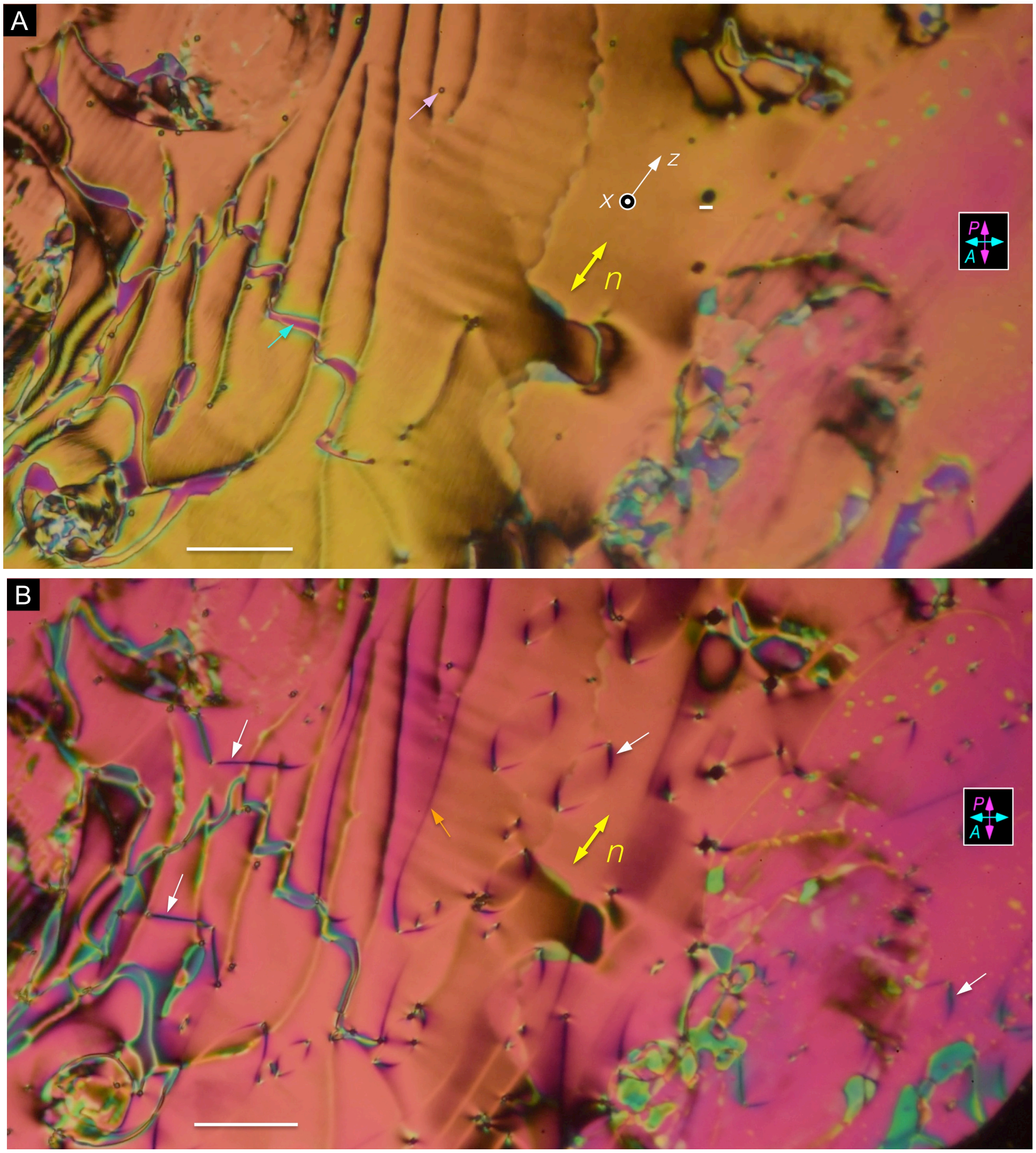

Figure S6: Typical DTLM images of the $t = 11\ \mu$m, planar-aligned, in-plane field cell with $E = 0$. (**A**) N phase ($T = 140$°C). (B) N_F phase ($T = 120$°C). The director ***n*** is generally along the buffing direction ***z***, here making an angle of about 45° with the crossed polarizer and analyzer but giving good extinction when rotated so that ***n*** is along the polarizer or analyzer direction. The linear defects in the texture are π director disclinations and surface memory reorientations [28] trapped at the I–N transition. The director is generally uniform along ***x***, the normal to the plates, except for a few twisted areas [cyan arrow in (**A**)]. Several silica spheres of 4μm optical diameter for size reference (***Fig. S10***) are also visible [pink arrow in (**A**)]. White arrows in (**B**) indicate typical Pure Polarization Reversal (PPR) lines, which upon cooling from the N phase, mediate the reversal of *P* in space; the orange arrow points to a PPR line running nearly along ***n***. The N and N_F textures are locally smooth (optically featureless), except near the phase transition as illustrated in ***Figures S8,9***. The distribution of ***P*** along +***z*** and -***z*** is roughly equal. Scale bar = 70 μm.

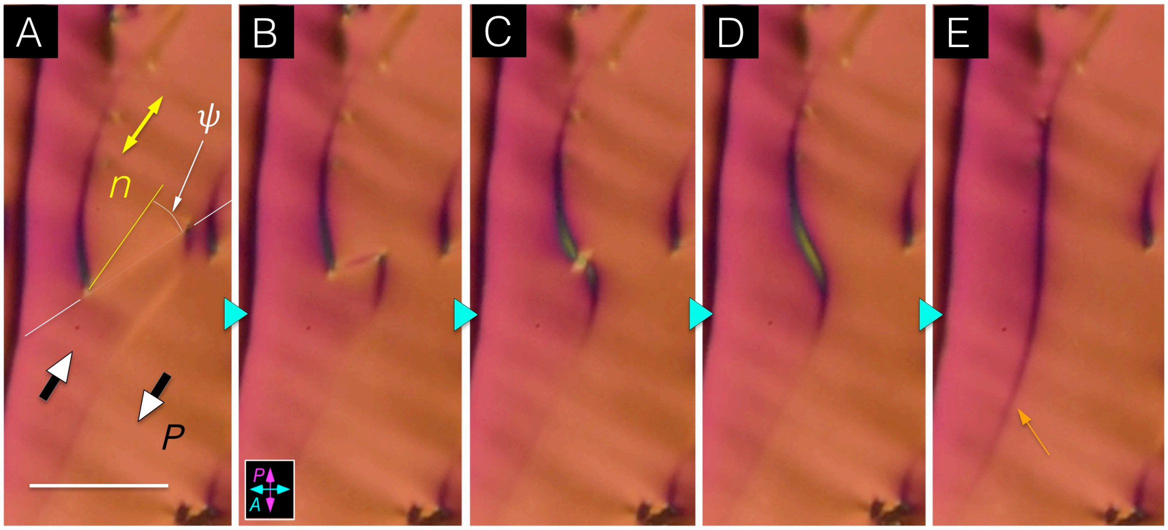

Figure S7: (**A**→**E**) Evolution of the PPR domain boundary wall indicated by the orange arrow in (**E**) and in ***Figure S6B*** during the coarsening (***Figures 1,S8,S9***). Ferroelectric polarization directions are shown in (**A**). Within the interior of the wall, $\boldsymbol{n}$ is always parallel to the wall direction, whereas outside of the wall $\boldsymbol{n}$ has its macroscopic orientation along the buffing (yellow line), maintained by polarization charge stiffening. (**A**→**C**) This difference makes increasing the angle ψ between the wall and $\boldsymbol{n}$ increasingly costly in energy, creating a line tension that shortens wall segments as ψ increases. $T = 120$ºC. Scale bar = 40 μm.

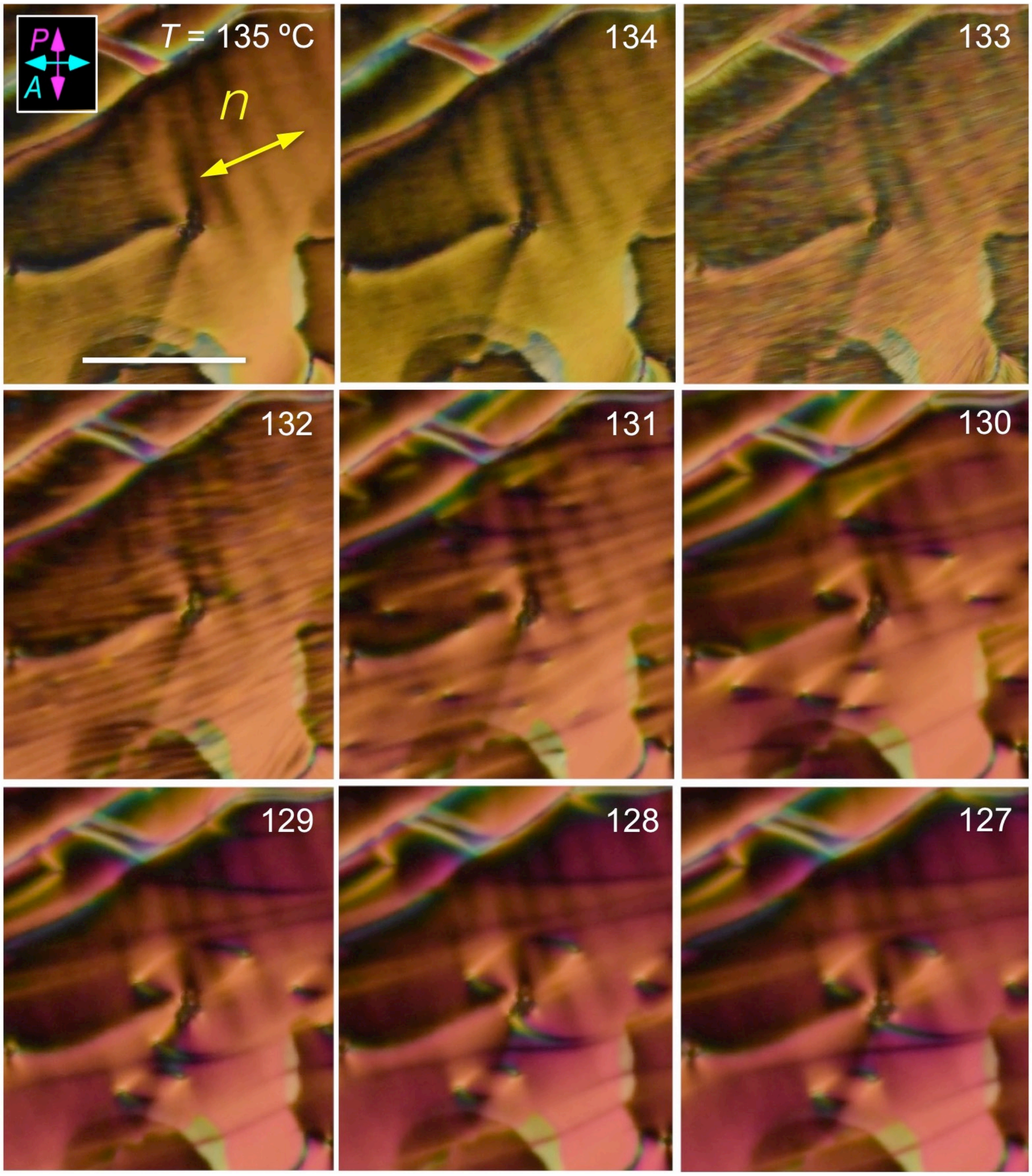

Figure S8: DTLM images obtained while cooling through the N–N_F transition, showing anisotropic polar correlations growing in size in the N phase as the transition to the N_F at T = 133ºC is approached. This pattern of bright and dark domains exhibits dynamic fluctuations in the N phase. We propose that in the N phase this extreme anisotropy is a result of the suppression of fluctuations along z of the magnitude of $\boldsymbol{P}(\boldsymbol{r})$ by polarization charge (dipole-dipole) interactions. Below the transition, the texture is characterized by macroscopic ferroelectric domains bounded by pure polarization reversal (PPR) lines which move and coalesce to coarsen the texture. $t = 11$ μm. Scale bar = 80 μm.

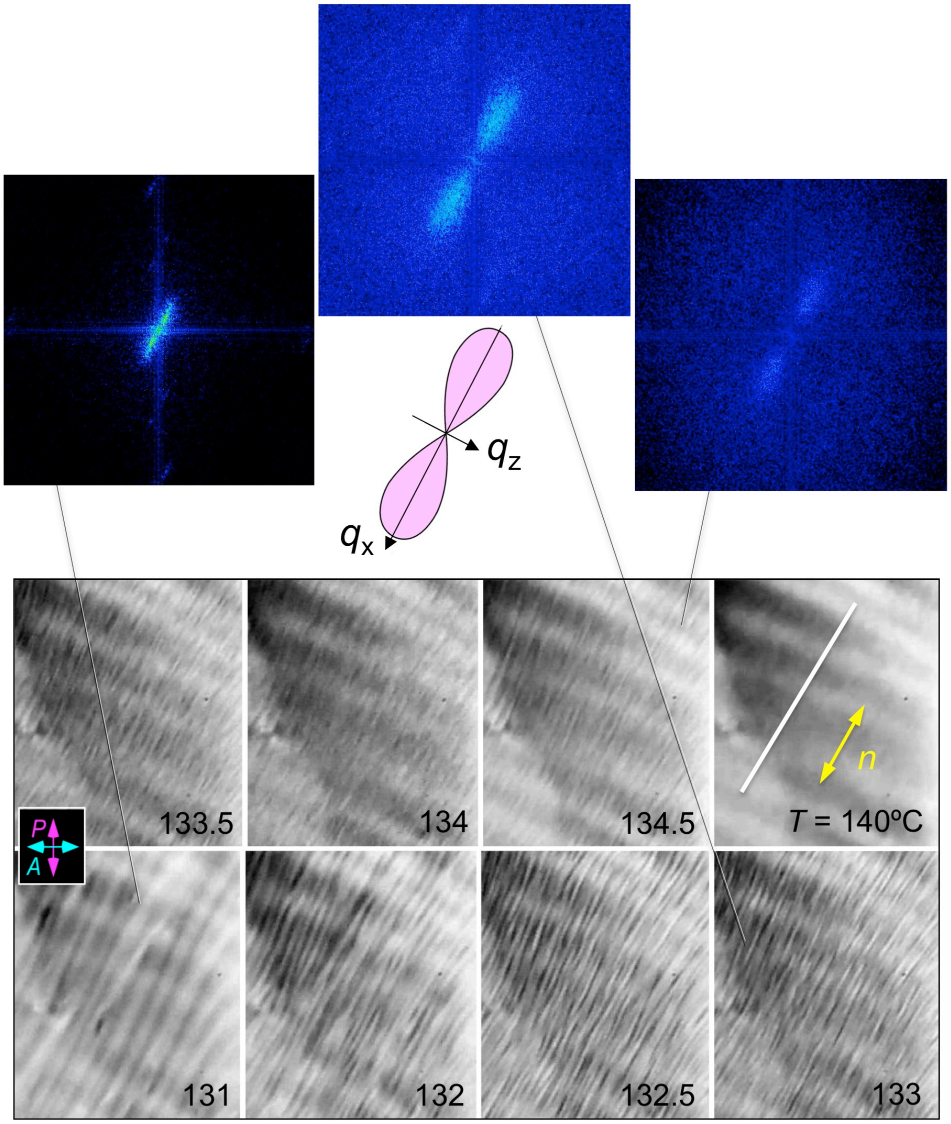

***Figure S9**:* Grayscale DTLM images of a sample area of the RM734 texture and selected optical Fourier transforms near the N–N_F phase transition. In the N phase (T < 133ºC), the images represent an optical resolution integration of the birefringent phase accumulated by the light upon traversing the sample thickness, t. The resulting averaging captures the extension of the domain shape along $\boldsymbol{n}$, which gives the wing-shaped optical Fourier transform (OFT). The pink inset shows a contour of the intensity that would be scattered by polarization fluctuations in the x-ray region given by

$$\chi_P(\boldsymbol{q}) \propto \langle P_z(\boldsymbol{q})P_z(\boldsymbol{q})^* \rangle \ \propto [\tau(T)(1+\xi(T)^2q^2)+(2\pi/\varepsilon)(q_z/q)^2]^{-1}$$

as discussed in the text. This susceptibility describes the anisotropy in the polarization fluctuations in the N phase generated by polarization charge interactions. The favorable qualitative comparison with the T = 133ºC OFT suggests that charge stabilization is an important factor governing fluctuations in the N phase. In the N_F phase, PPR lines (domain walls) extend long distances along $\boldsymbol{n}$, producing sharp intensity bands in the Fourier transform image that are normal to $\boldsymbol{n}$. These textures suggest that resonant and non-resonant SAXS and WAXS could be effective probes of $\chi_P(\boldsymbol{q})$, as well as of the transverse fluctuations of $\boldsymbol{P}(\boldsymbol{r})$. t = 11 μm. Scale bar = 80 μm.

N–N$_F$ phase coexistence upon melting the N$_F$ – Slow heating of RM734 through the N$_F$–N transition results in a distinctive melting process in which the nematic appears as extended, flexible filaments in a background of N$_F$. Examples of these filaments are shown in ***Fig. S10***, where a nematic front is moving in from the right as the temperature increases slowly. The filaments, which have diameters D_f ~2-4 μm, grow in from the right, where their density is largest. The nematic filaments penetrate the N$_F$ phase but do not otherwise disturb it, with the N$_F$ maintaining its alignment and birefringence independent of any nearby filaments. The nematic alignment inside each filament appears to be uniform, with the director oriented parallel to the filament axis, so that the filaments can generally be seen with high contrast, except where they are aligned parallel to the N$_F$ background field. As with the domain walls in the N$_F$ phase, the nematic filaments lose optical contrast when oriented parallel to the director field of the background N$_F$ phase. The orientation of the filaments does not appear to be strongly coupled to the N$_F$ director field and varies greatly across the sample. These filaments show that the N and N$_F$ phases can coexist as distinct states, at least meta-stably.

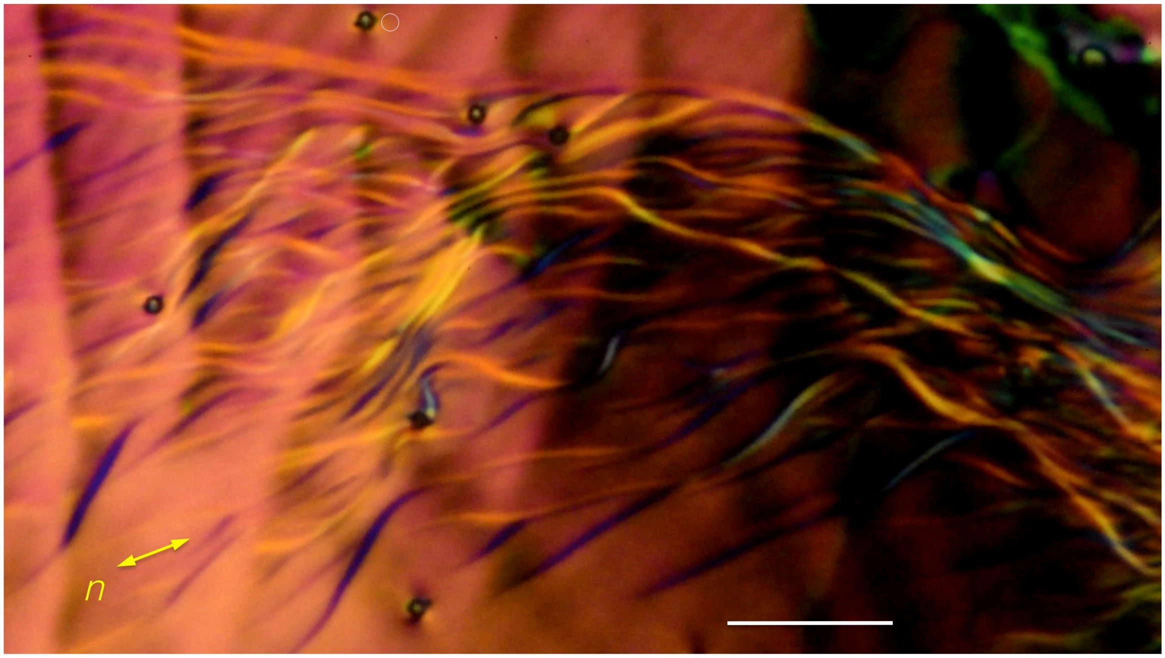

Figure S10: Filamentous domains of the N phase moving into the N$_F$ phase upon heating through the N–N$_F$ transition near T = 130 ºC in a t = 11 μm cell. The N filaments grow in from the right. The filaments lose their optical contrast when they are oriented parallel to the director of the background (orange) N$_F$ phase, indicating that, as in the PPR walls, the director inside the filaments is oriented parallel to the filament boundary. This phase coexistence is evidence that the N–N$_F$ transition is first order.)]. Scale bar = 40 μm. Occasional silica spheres are visible in many of the main text and SI Appendix photos, for visual size reference (***Figs. 4, S6,S11***). Their apparent diameter is ~4 μm, the diameter of the white circle at the top of this image.

Section S4 – Combining PPR and PnD Domain Walls

The combination of π-PPR and π-***Pn***D lines creates a novel hybrid defect line unique to the N_F phase. ***Fig. S11A*** shows two PPR lines separating domains of opposite ***P***, with a small field beginning to reverse the central domain. The PPR lines are barely visible, as noted in ***Fig. S7***, but increased field strength reverses the central domain, making it visible and leaving unswitched (green) sheets ~ξ_P thick at the surfaces. Then, at sufficiently high field, in a process analogous to the "order reconstruction" of confined splay in a nematic [29,30], pink holes open up in these sheets (***Fig. S11B***). This nucleation and the resulting movement of π-***Pn***D lines to the boundaries of the central domain, leave it completely pink, i.e., uniformly switched with ***P*** now along ***E***, and now identical in structure to the outer area, separated from it by the hybrid π-PPR/ π-***Pn***D 2π-walls, now visible because of their ***Pn***D component. The cross-sectional structure in ***Fig. S11C*** shows the 360° reorientation of ***P*** on passing through the wall and that in an applied field, such walls have energy in excess of that of the surrounding areas. This energy difference increases with field, which confines the 180° ***Pn***D reorientation of the wall to a central sheet of width $\xi_E = \sqrt{K/PE}$, a high energy director configuration in a sea of uniform orientation. At sufficiently high field, "order reconstruction" holes [29,30] nucleate in the sheet, inducing gaps in the wall such that it recedes (***Fig. S11D-G***). This process is one-way: once the wall recedes, the area vacated shows no memory of it when the field is reversed. As a result, the PPR lines in a newly cooled cell tend to disappear by nucleation and movement of gaps when exposed to a changing field. The comparisons above of polar vs. dielectric response to field also apply to this process.

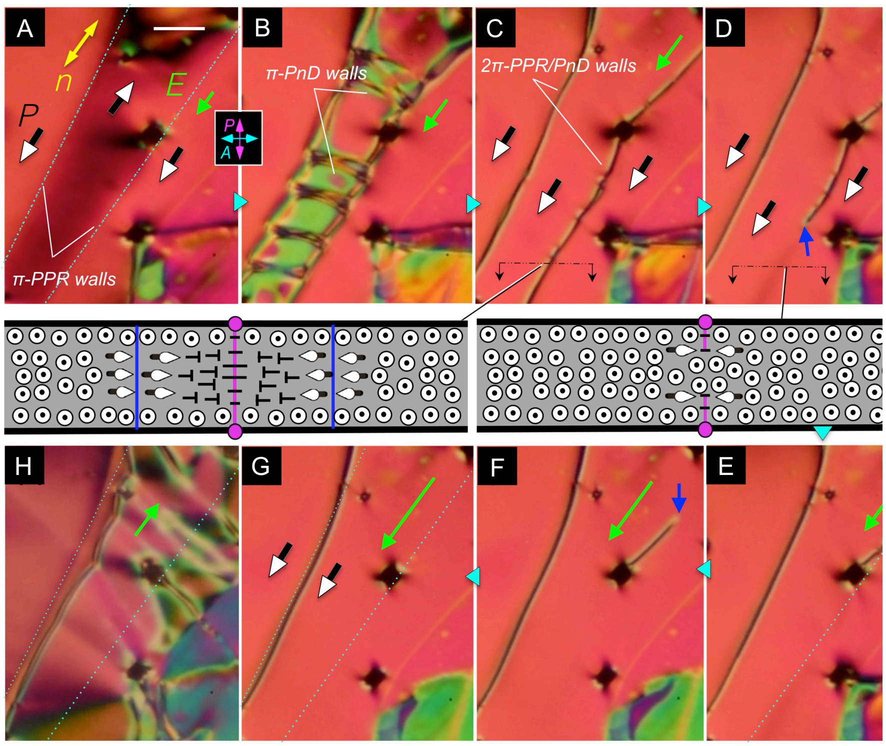

Figure S11: Polarization-reversal and hysteresis: (**A**) Domains with opposite polarization grown in at the N–N_F transition (central domain up, outer domains down) separated by pure polarization reversal (PPR) π-walls. (**B,C**) Field-induced reversal of the central domain by bulk and surface reorientations, leaving it separated from the outer domains by composite 2π-PPR/***Pn***D walls. (**D–G**) Gap nucleation in, and shrinking of, 2π-PPR/***Pn***D walls, induced by increased field. Since the walls have higher energy than the uniform state that replaces them, they are squeezed out by the field (blue arrows) and do not regrow upon decreasing the field, a hysteretic response. (**H**) A second field reversal produces a bend instability in the director field like that shown in ***Figure 5***. The sections in **C** and **D** show the 2π-PPR/***Pn***D wall structure and its squeezed-out remnant respectively. Scale bar = 25 μm.

***Section S5** – Splay in the N and N_F Textures*

The evolution of the cell upon cooling into the N_F phase, ending, apart from the presence of the domain walls in ***Fig. 1***, in textures that are optically smooth with close to the same birefringence as the nematic, suggests that there are no sub-resolution orientational modulations or non-uniformities that would reduce the birefringence of the equilibrium $\mathbf{n}(\mathbf{r}),\mathbf{P}(\mathbf{r})$ fields of the N_F phase below that of the N phase. However, the polar symmetry of the N_F phase implies a polar orientational distribution of the molecules, which, because of the lack of reflection symmetry of their steric shape, should generate a tendency for director splay in the N_F phase that is correlated with the direction of $\mathbf{P}(\mathbf{r})$, like that of flower stems in a bouquet, with $\mathbf{P}(\mathbf{r})$ indicating the growth direction [31]. This effect has been observed in calamitic chiral smectics [32], and in polar-ordered smectic bent-core B2 phases that are ferroelectric (SmC_SP_F) [33]. The latter break up into structures of linear splay domains, an instability which generates the B7 phases, for example [34]. Such structures appear only above a threshold value of preferred splay amplitude because filling 3D space with splay always requires some sort of non-splayed or oppositely-splayed defects that cost extra energy [19,35]. Below this threshold, the splay is simply frustrated in domains of uniform orientation. In some situations, however, splay is required, such as in the $\mathbf{n}(\mathbf{r}),\mathbf{P}(\mathbf{r})$ field around the air bubbles in ***Fig. 5C*** and ***Fig. S12***, and around and inside the P-reversal domains in ***Fig. 1D*** and ***Fig. S5***. Here different signs of polarization splay are required to make the two 180° wedge defects on opposite sides of the bubble (red dots), and at opposite ends of the polarization domains. By symmetry, these opposing structures must be different. However, they are in fact very similar in appearance, implying that the preference for a particular sign of splay does not significantly affect their structure. The N_F phase therefore appears to have large polarization but a weak tendency for splay, indicating a small splay flexoelectric coefficient [36] that is perhaps a result of its molecular-scale organization, discussed in the text and in ***Section S10***.

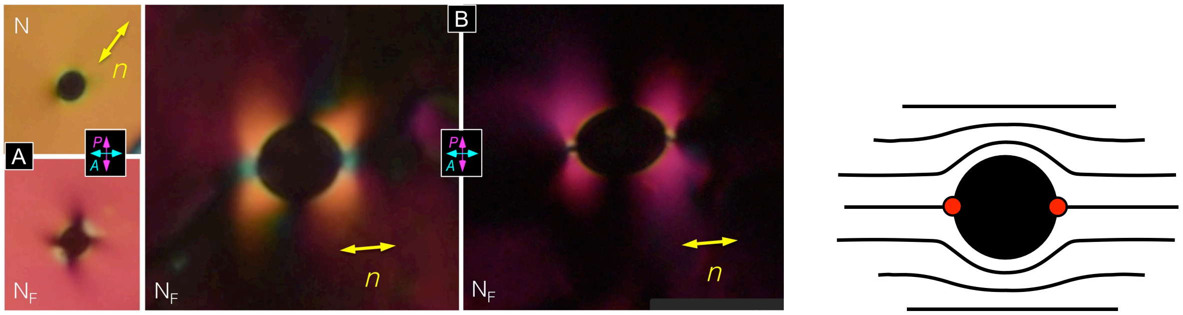

Figure S12: DTLM images of textures around an air bubble in the t = 11 μm cell. Orientational anchoring at the bubble /LC interface forces the director $\mathbf{n}$ to be tangent to the interface, so that it bends along the bubble perimeter. (**A**) A 30 μm-diameter bubble in the N and N_F phase. (**B**) 35 μm-diameter (**left**) and 60 μm-wide (**right**) bubbles in the N_F phases. Comparing the N and N_F textures shows that the tangential surface anchoring is stronger in the N_F phase and that the director bend persists to much larger distances from the bubble surface. This is due to the competition between strong polarization charge-generated splay rigidity and normal bend rigidity in the N_F phase, making splay deformations very gradual. Here π wedge disclinations (red dots in the sketch at right) stabilize required splay near the bubble.

***Section S6** – Sandwich Cell Electro-Optics & the Splay-Bend Freedericksz Transition*

The N_F phase exhibits characteristic high-polarization ferroelectric LC behavior, illustrated in ***Fig. S13***, which shows a sequence of DTLM images of RM734 in a conventional, transparent capacitor cell t = 4.5 μm thick, with antiparallel-buffed polyimide-coated ITO electrodes that give unidirectional planar alignment with a few degrees of pretilt. The sample temperature is held near the N–N_F transition, a small, in-plane temperature gradient ensuring that part of the sample is in the N_F phase while the rest is in the N phase, with a wide transition region (demarcated by white and black dashed lines in ***Fig. S13A***) where the two phases form overlapping wedges (sketched in ***Fig. 8C***) and the birefringence is lower. In the N phase, the LC responds dielectrically to an applied 1 kHz triangle wave voltage of amplitude V_p, resulting in a decrease of birefringence Δn, beginning at $V_p \sim 1.5$ V. For $V_p \sim 3$ V, the birefringent optical retardance Δnt is reduced from 900 nm to 300 nm [37], corresponding to $\theta(V_p)$ increasing from $\theta \sim 0^o$ to $\theta \sim 54^o$, a substantial rotation of $\boldsymbol{n}$ toward the field direction. However, the net birefringence increases continuously through the wedge region as the thickness of the N layer decreases (***Fig. S13C***). In contrast, the part of the cell in the N_F phase exhibits no observable change in birefringence in this voltage range. This behavior can be understood on the basis of the "block polarization" model developed to explain V-shaped switching in high-polarization FLCs [15,38,39]. In this model, polarization charge induced by reorientation of $\boldsymbol{P}$ completely cancels E in the LC and the polarization direction is electrostatically controlled. Insulating layers without reorienting polarization at the LC/glass interfaces such as the polymer alignment layer, charge depletion in the ITO, and bound polarization at the surface, are accounted for as capacitive elements with a net capacitance per unit area, C. With these assumptions, the orientation of the polarization field as a function of applied voltage V is given by $\sin\theta(V) = V/V_{sat}$, where $V_{sat} = P/C$ [15,38,39], showing that when P is large, a large applied voltage is required to achieve substantial reorientation of $\boldsymbol{n},\boldsymbol{P}$. This N_F cell can be compared with the bent-core $SmAP_F$ ITO electro-optic cell of Ref [15], where a saturation voltage $V_{sat} = 15$ V was measured for material with $P = 0.85$ $\mu C/cm^2$, leading us to expect $V_{sat} \sim 100$ V for LC in the N_F phase with $P \sim 6$ $\mu C/cm^2$ in a cell with 50 nm-thick insulating layers with $\varepsilon = 5$ at the two cell boundaries. It is therefore not surprising that the N_F phase shows very little response in the applied voltage range explored here. In a further experiment, we cooled through the N–N_F transition while continuously applying a $V_p \sim 16$ V triangle wave, observing Freedericksz-type reorientation to a homeotropic state in the N phase and the reimposition of planar alignment by polarization stabilization in the N_F phase (see ***Fig. S14***).

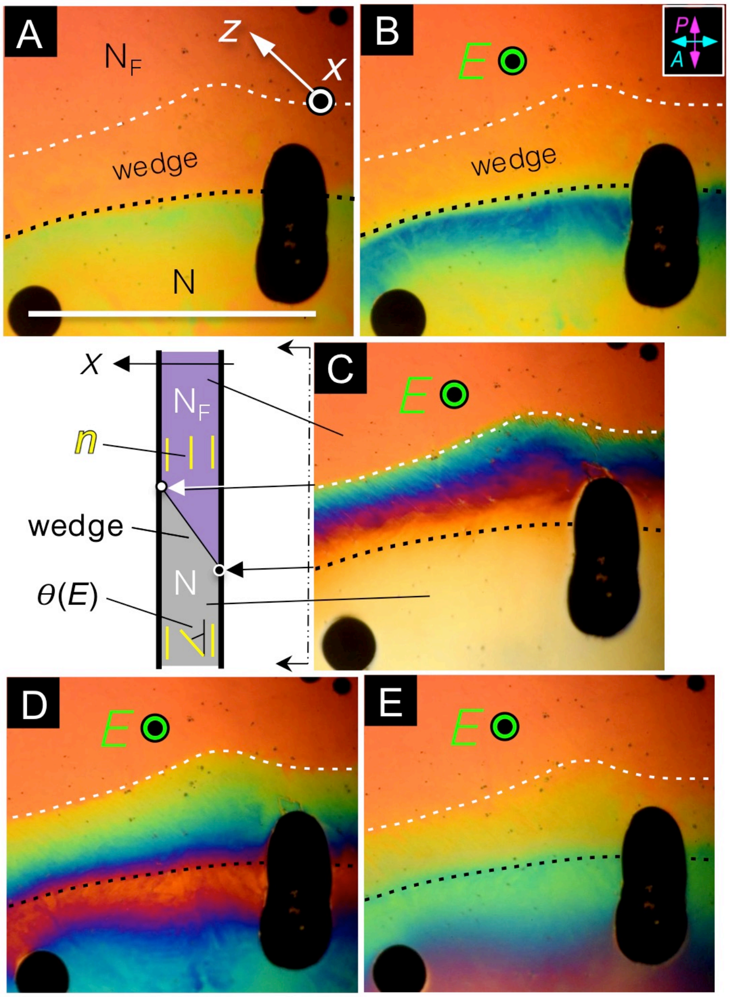

Figure S13: Splay-bend Freedericksz transition of RM734. The LC is in a $t = 4.5\ \mu$m-thick transparent capacitor cell with polyimide-coated ITO electrodes antiparallel buffed along z giving unidirectional planar alignment with a few degrees of pretilt. The temperature increases by several degrees from top to bottom of the image, creating a wedge-like interface between the N_F and N phases. (**A**) Planar aligned RM734 with no field applied. The director away from the phase boundary is oriented essentially parallel to the glass ($\theta \approx 0^o$). (**B**) In a 1 kHz triangle wave voltage of amplitude $V_p \sim 1$ V, the director begins to align with the field, lowering Δn. (**C**) With $V_p \sim 3$ V, $\theta(V_p)$ is increased to $\theta \approx 54^o$ in the lower N part of the cell. The N_F region, in contrast, shows no change in Δn with field. The change in birefringence across the wedge reflects the different director configurations obtaining in the N and N_F phase tongues. (**D**) The return to planar alignment ($\theta = 0^o$) upon reducing the field shows some hysteresis, with the region of the N phase nearest the N_F retaining a slightly larger tilt θ. (**E**) Texture on reducing the voltage to $V_p \sim 1$ V, as in (**B**). Scale bar = 1 mm. The black regions are air bubbles.

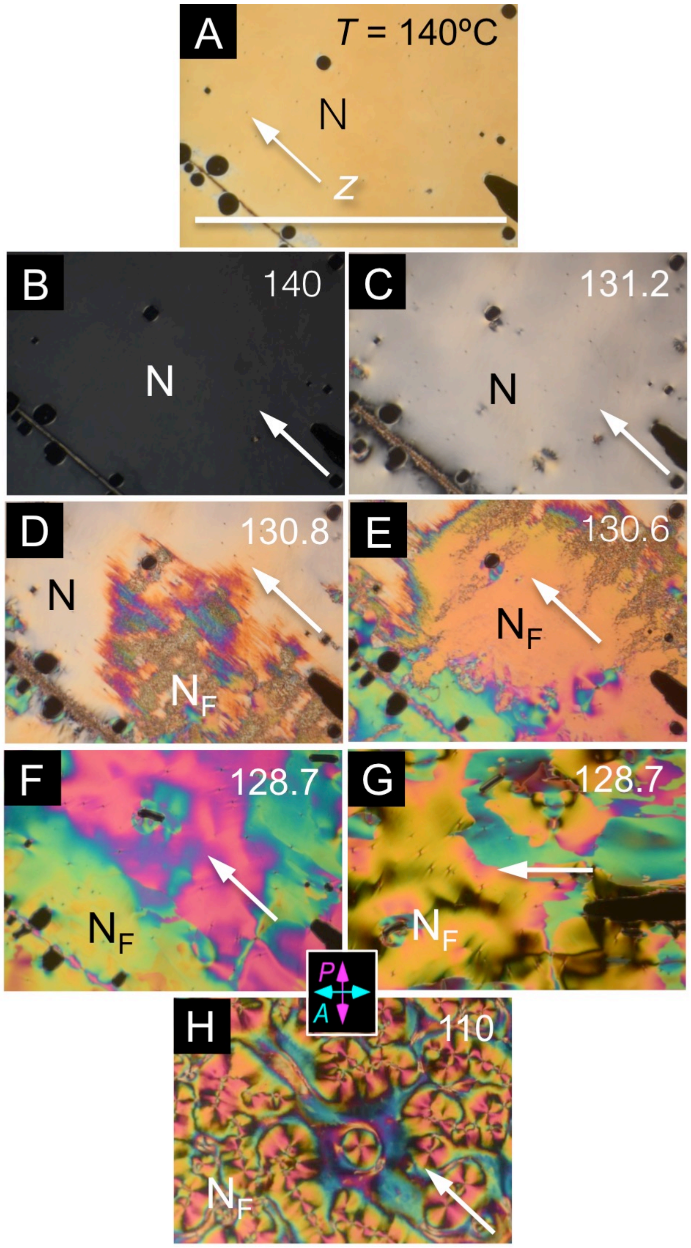

Figure S14: Splay-bend Freedericksz texture cooling sequence of RM734 in the ITO sandwich cell of *Fig. 8*. (**A**) Planar-aligned, with no field applied. (**B–H**) This cell is cooled through the N–N_F transition while continuously applying a V_p ~ 16 V, 500 Hz triangle wave, well above the Freedericksz threshold in the N phase. (**B**) The N phase with the field applied starts at θ ~ 85° (above threshold and nearly homeotropic) at T = 140°C but (**C**) on approaching the transition, θ decreases to θ ~ 80° near T = 131°C, where (**D**) the N_F phase comes in via irregular domain boundaries that (**E**) anneal into an ordered θ ~ 0° planar-aligned N_F domain (orange birefringence color). However, (**F–G**) as T is lowered to T = 120°C this field induces a defected pattern of increased θ, a birefringent texture that is permanently written when the field is removed. This process must involve breakdown of the interfacial capacitors discussed in the text. Texture (**H**) is obtained with a V_p ~ 16 V, 1 Hz triangle wave. Scale bar = 1 mm.

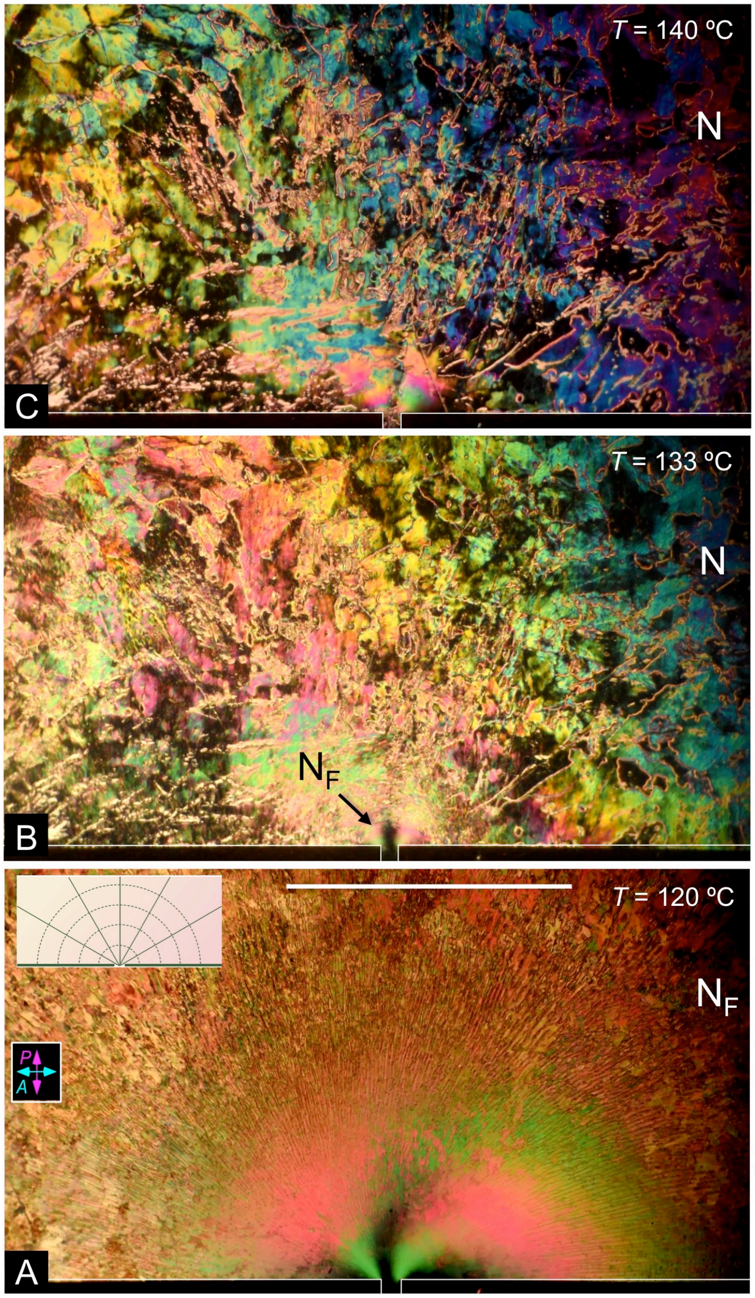

Figure S15: Field-induced flow in RM734 vs. temperature. DTLM images of a t = 10 μm-thick planar - aligned cell of RM734 between untreated glass plates. The black bars at the bottom of each image are two evaporated gold electrodes on one of the plates, separated by a d = 60 μm gap, outlined in white for clarity. A V_p = 3 V peak 0.1 Hz square-wave voltage is applied to the electrodes in all images. Only the upper portion of the electrodes and cell are shown. The cell is shown at three different temperatures: (**A**) T = 120ºC in the N_F phase. The applied voltage drives a pattern of defect motion and fluid flow over the entire image, with the defect velocity $\boldsymbol{v}(\boldsymbol{r})$ vectorially parallel to the applied field, $\boldsymbol{E}(\boldsymbol{r})$, on circular arcs centered on the electrode gap. The graph shows the E-field lines (dashed) obtained by solving the Laplace equation. This image is taken at the instant of field reversal, where the resulting polarization reversal generates a periodic array of bend domain walls normal to the director, as in ***Fig. 5A***, radial in this case (inset solid lines). (**B**) T = 133 ºC. Most of the cell area is in the N phase and not flowing. The region near the electrode gap where the field is the highest is the last to remain in the N_F and is still flowing. (**C**) T = 140ºC. The LC is entirely in the N phase, with a Schlieren texture, and there is no observable flow, showing that the defect motion and flow are features of the N_F phase (***Fig. 6***). Scale bar = 1 mm. See also ***Fig. S16***.

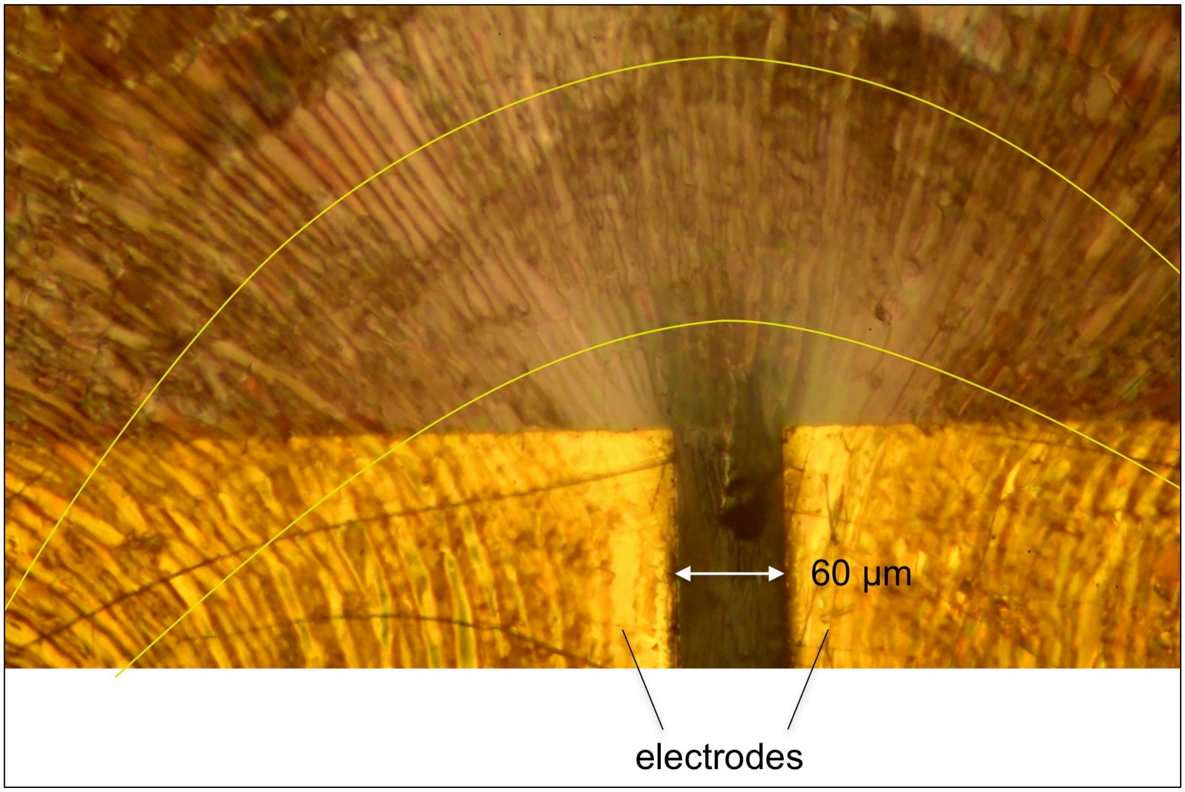

Figure S16: Reflected light microscope image of the cell in ***Fig. S15***, a t = 10 μm-thick planar-aligned cell of RM734 between untreated glass plates, with two evaporated gold electrodes separated by a d = 60 μm gap on one of the plates. This image was captured at T = 120 ºC at the instant of applied field reversal, where the resulting polarization reversal generates a periodic array of bend domain walls locally normal to the reversing $\boldsymbol{E}$ and to the director field $\boldsymbol{n}(\boldsymbol{r})$, as in ***Fig. 5A***. At the top of the image, the lines are approximately radial as in ***Fig. S15A***. This image shows a remarkable feature of the N_F electro-optics: even the weak applied field that penetrates over the electrodes is strong enough to produce polarization reversal in the LC. The yellow lines are along the director field.

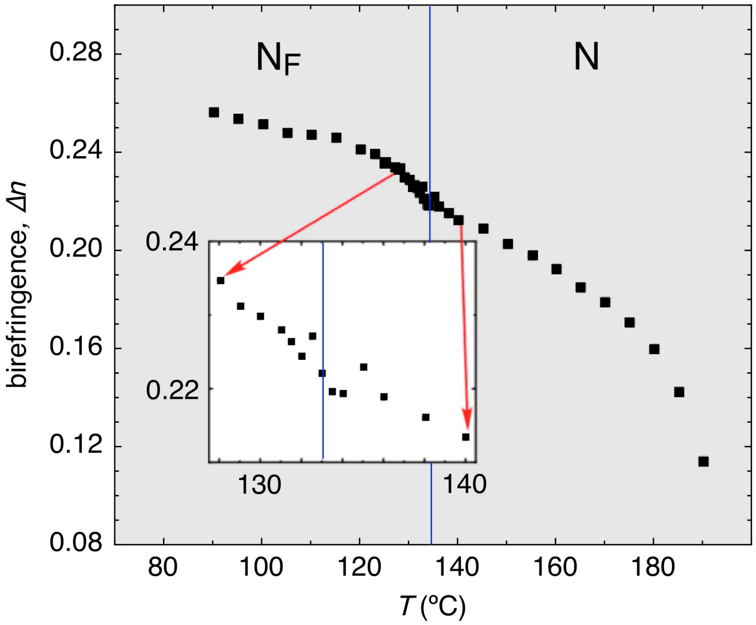

Figure S17: Birefringence of RM734, measured in a t = 3.5 μm-thick, planar-aligned cell using a Berek compensator.

SECTION S9 – ATOMISTIC SIMULATION METHODOLOGY

Force Field – All molecular dynamics (MD) simulations were conducted using the Atomistic Polarizable Potentials for Liquids, Electrolytes and Polymers (APPLE&P) force field [40,41]. The parameters for atomic polarizabilities and repulsion-dispersion interactions were taken from the APPLE&P database without modification, while atomic charges were fitted to reproduce the electrostatic field around all of the molecular fragments as obtained from MP2/aug-cc-pVDZ quantum chemistry calculations using Gaussian 16 software [42,43]. The parameters for missing dihedral potentials were obtained by fitting conformational energy scans obtained from DFT calculations at the M052X/aug-cc-pVDZ level of theory [44]. A non-polarizable version of the force field was also used, with the atomic polarizabilities set to zero and all other parameters kept the same as in the polarizable version.

Simulation Parameters – The simulations were carried out using the WMI-MD simulation package (http://www.wasatchmolecular.com). In these simulations, all covalent bonds were constrained using the SHAKE algorithm [45]. The potential energy of bond-angle bending, out-of-plane bending, and dihedral angles was described with harmonic potentials or cosine series expansions [40]. The van der Waals interactions were calculated within a cut-off distance of 12.0 Å, with a smooth tapering to zero starting from 11.5 Å. The charge-charge and charge-induced dipole interactions were calculated using Ewald summation [46]. The induced dipole-induced dipole interactions were truncated at 12.0 Å. To avoid the polarization 'catastrophe', a Thole screening parameter of 0.2 was used for small separations between induced dipoles [47]. A multiple time step integration approach was used to enhance computation efficiency. A 0.5 fs time step was used for the calculation of valence interactions, including those involving bonds (SHAKE), bond angle bending, dihedral angles, and out-of-plane deformations. The short-range, non-bonded interactions (with 7.0 Å radius) were calculated every 1.5 fs, while a time step of 3.0 fs was employed for the remaining non-bonded interactions and the reciprocal part of the Ewald summation.

System Initialization and Simulation Protocol – Simulation cells were prepared with two different initial configurations of the molecules: (***i***) ***POLAR*** (*POL* – all molecules oriented along the +z direction), and (***ii***) ***NONPOLAR*** (*NONPOL* – equal numbers of molecules oriented along the +z and –z directions). Initially, the 384 molecules were positioned on a relatively low-density lattice with simulation cell dimensions of 150 Å in the x and y directions and 70 Å in the z direction. A 630 ps compression simulation was then conducted to achieve a mass density of ~1.0 g/cm^3 (comparable to typical thermotropic liquid crystal mass densities), with the z-dimension of the simulation cell fixed at 70 Å, and with a biasing potential applied to the ends of the mesogens to preserve their orientation during the initial equilibration stage. The biasing potentials were then removed and further equilibration runs 6 ns in duration and production runs in excess of 20 ns were carried out. All simulations were conducted in the NPT (isobaric, isothermal) ensemble with the z-dimension of the cell fixed and the x and y dimensions allowed to vary to maintain a constant pressure of 1 atm (NPT-XY ensemble). Each system was simulated at 110ºC, 130ºC, 150ºC, and 180ºC, temperatures spanning the N_F - N phase transition, using polarizable and non-polarizable force fields. The temperature and pressure were controlled with the Nose-Hoover thermostat and barostat [48]. The same simulation protocol was used to model the cyano-terminated mesogen RM734-CN [1,2].

SECTION S10 – ATOMISTIC SIMULATION RESULTS

Order parameters – An instantaneous configuration of the *POL* system at T = 130 °C is shown in ***Fig. S18***, revealing that the system retains a high degree of orientational order at this temperature. To quantify nematic orientational order in this system, we measure the traceless, symmetric nematic ordering tensor , where *I* is the identity matrix, and where the sum ranges over all molecules. The scalar nematic order parameter *S* corresponds to the largest eigenvalue of the time-averaged ordering tensor , and the biaxial

order parameter B is defined as the difference between the two smallest eigenvalues. Polar order is assessed by measuring the (vector) polar order parameter, from which a scalar polar order parameter can be obtained. For the *POL* simulation of the polarizable model at 130 °C, we measure a large nematic order parameter, S = 0.787 ± 0.009, and a nearly saturated polar order parameter, Π = 0.924 ± 0.003, with negligible biaxiality (B = 0.013 ± 0.002). Moreover, the polar order parameter $\boldsymbol{P}$ is colinear with $\boldsymbol{n}$, the principal eigenvector of . Given that there appear to be no long-range positional correlations (as shown below), the simulated state appears to be a uniaxial ferroelectric nematic (N_F) phase.

It is interesting to compare these results with those from the *NONPOLAR* simulation under the same conditions (T = 130 °C, polarizable molecular model). The nematic order parameter in this case is S = 0.78 ± 0.02, quite similar to that of the polar system, while the polar and biaxial order parameters are small (P = 0.013 ± 0.004, B = 0.028 ± 0.003), as expected for a conventional uniaxial nematic (N) state. The fact that the magnitude of S is nearly the same in the *POL* and *NONPOL* states is generally consistent with the experimental observation that the birefringence does not change significantly through the N–N_F transition. The simulated mass density at T = 130ºC is δ = 1.3 g/cm^3.

Ferroelectric polarization density **–** The measured spontaneous ferroelectric polarization density P in the N_F phase of RM734 is large, increasing with decreasing temperature below the N–N_F transition to a saturation value of around P = 6 μC/cm^2 (***Fig. 3***). This implies a high degree of polar order in the N_F phase, that can be further quantified by comparison with the polarization computed by simulation.

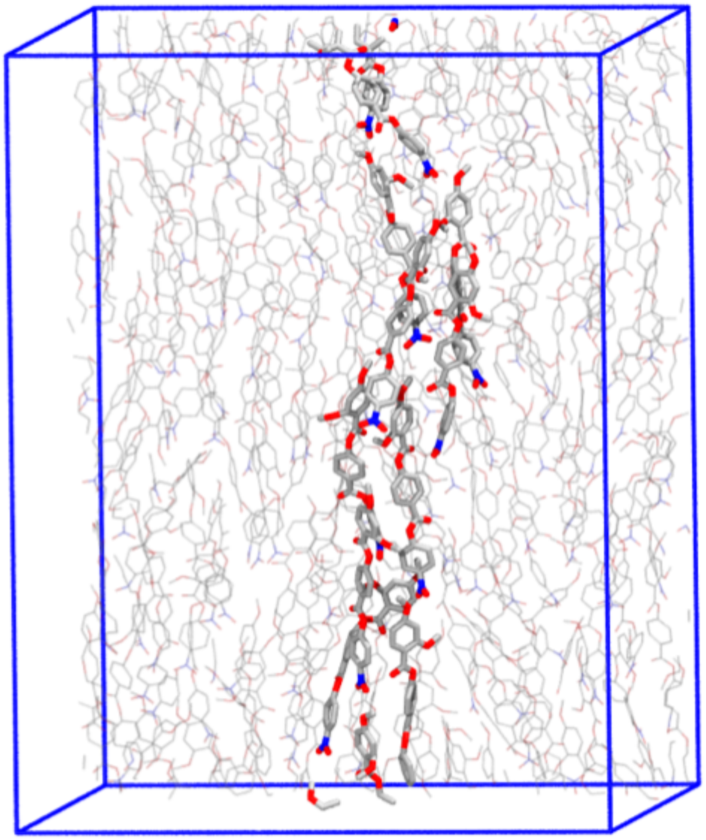

Figure S18: (A) Instantaneous configuration from the *POL* atomistic MD simulation system of 384 RM734 molecules at T = 130°C, employing a polarizable molecular model. The system was initialized in the *POL* state, and equilibrates to a density δ = 1.3 g/cm^3 with a high degree of nematic (S = 0.79) and polar (Π = 0.92) orientational order. End-to-end flips are not observed. The vertical cell dimension is 70 Å.

As noted above, RM734 has a large electric dipole moment p = 11.4 D, as determined from quantum chemistry calculations at the B3LYP/6-31G* level of theory (***Fig. S19***). Higher-level quantum chemistry calculations were used to assign site charges to atom sites and lone pair electron sites in the molecular mechanics model used in our atomistic simulations. The resulting static site charges, shown in ***Fig. S20,*** are also consistent with a molecular dipole moment of around 11 D (computed using polarization , where $\boldsymbol{r}_i$ and q_i are the site positions and charges, and the sum ranges over all n sites in the molecule). Note that the dipole mo-

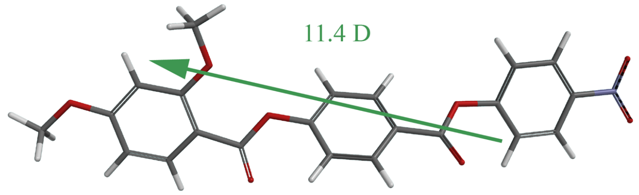

Figure S19: Geometry-optimized structure of RM734 computed at the B3LYP/6-31G* level of theory, showing the orientation of the 11.4 D molecular dipole moment (green arrow) for this specific molecular conformation. Other low-energy conformations have comparable dipole moments.

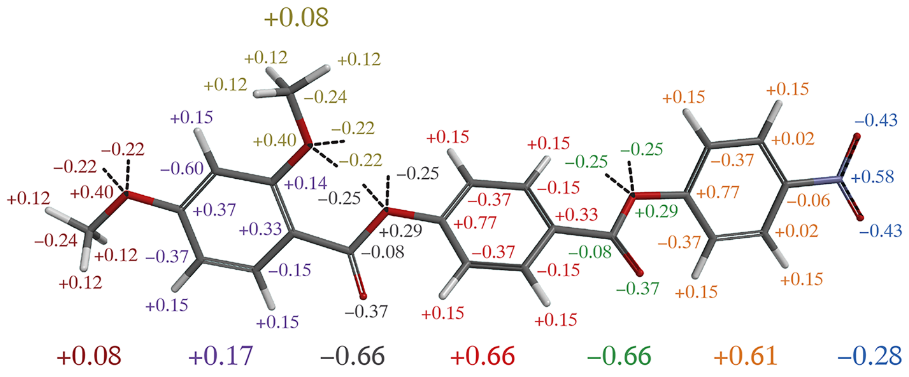

Figure S20: Static site charge distribution used in the atomistic simulations. The overall charges of specific functional groups, indicated in large type, show an alternation of group charges along the length of the molecule. The dashed lines correspond to lone-pair electrons.

ment has a weak dependence on molecular conformation, and that molecules sample an ensemble of low-energy conformations over the course of a simulation. For the *POL* simulation of the polarizable model at 130°C, we measure an average static molecular dipole moment (from static site charges) of magnitude = 11.24 ± 0.01 D. For the polarizable models, there is also an induced molecular dipole moment component, which has an average magnitude of = 1.46 ± 0.02 D, but has a nearly isotropic orientational distribution, so the average magnitude of the total molecular dipole moment (the sum of static and induced contributions) is nearly equal to thestatic contribution, = 11.20 ± 0.01 D. The fact that the induced molecular dipole has a nearly isotropic orientational distribution is a consequence of the boundary conditions, which ensure that the average electric field is zero at any point in th e system (there is no bound charge at the surface of the system, so the depolarization field vanishes), so the average magnitude of the induced dipole moment vector is close to zero, = 0.053 ± 0.009 D.

We can gain further insight by resolving the total ferrolectric polarization density into contributions from specific dipolar groups. To accomplish this, we employ a unique decomposition of charges into elementary charge-neutral dipolar groups (bonds and rings), as shown in ***Fig. S21***. Group dipoles can be further aggregated into functional groups, which are indicated by color coding in ***Fig. S21***, together with the dipole moments and average contributions to ferroelectric polarization density P_S of specific functional groups. The terminal nitro group and the ring to which it is attached are highly dipolar, and make a dominant (~ 64%) contribution to P_S. Four functional groups (nitro, nitro ring, central ring, and terminal methoxy) account for ~ 90% of the molecular polarization density.

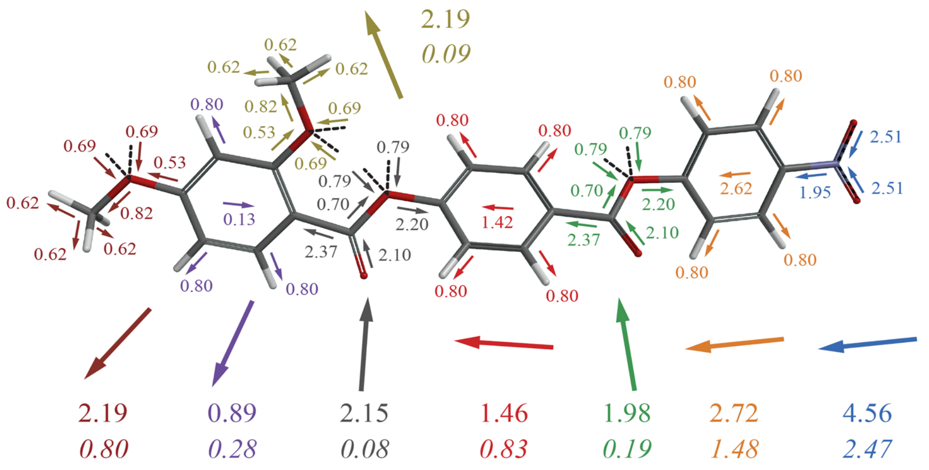

Figure S21: Decomposition of the static site charge distribution into group dipole contributions. Irreducible bond and ring dipole moments are shown as small arrows, where the numerical value is the dipole moment in Debye (D). The dipole moments of specific functional groups are also indicated (large arrows and large, non-italiced text). The numbers in italics are the average contributions of specific functional groups to the computed ferroelectric polarization density P_S, in units of μC/cm^2. The nitro group and the ring to which it is attached (the nitro ring) have the largest dipole moments, and together contribute 64% of the total polarization density. Four functional groups (nitro, nitro ring, central ring, and terminal methoxy) contribute 90% of the total polarization density. The ester groups and lateral methoxy possess substantial lateral dipole moments, which may contribute to intermolecular association.

We have calculated the average ferroelectric polarization density of the maximally polar equilibrium state of the *POL* simulation at 130°C from , where *V* is the system volume. For the *POLAR* simulation of the polarizable model, the polarization density magnitude = 6.17 ± 0.01 μC/cm^2, where only 0.13 ± 0.03 μC/cm^2 is due to the induced polarization. This calculated P_S is in quantitative agreement with the saturation polarization density *P* measured experimentally (***Fig. 3***). This value is also nearly the same as that obtained from the simple estimate given in the text assuming 100% polar order, as might be expected given the high value of the *POL* system polar order parameter given above, Π = 0.924 at T = 110ºC. The remanant orientational disorder is due to small orientation fluctuations about ***n***, basically all that is allowed in the *POL* system. Consequently, Π depends only weakly on T, with the MD giving = 6.170 ± 0.008 μC/cm^2 at T = 130ºC and = 6.368 ± 0.002 μC/cm^2 at T = 110ºC.

An important inference of this agreement with the N_F phase experimental value is that at low *T* RM734 essentially becomes the *POL* system, i.e. is a polar nematic with no molecular flips, and remnant polar disorder that is strictly short ranged (~few molecule) small angle orientation fluctuations about the director. At higher temperatures *P* decreases in the N_F phase because of the growth of longer length scale fluctuations and disordering modes. But these disappear upon cooling to the saturated state at low *T*, where the fluctuations in the N_F become consistent with those allowed in the nanoscale volume and periodic boundary conditions of the *POL* simulation. On the other hand, the simulation volume is chosen to be large enough to observe the local molecular packing driven by specific intermolecular interactions, discussed next.

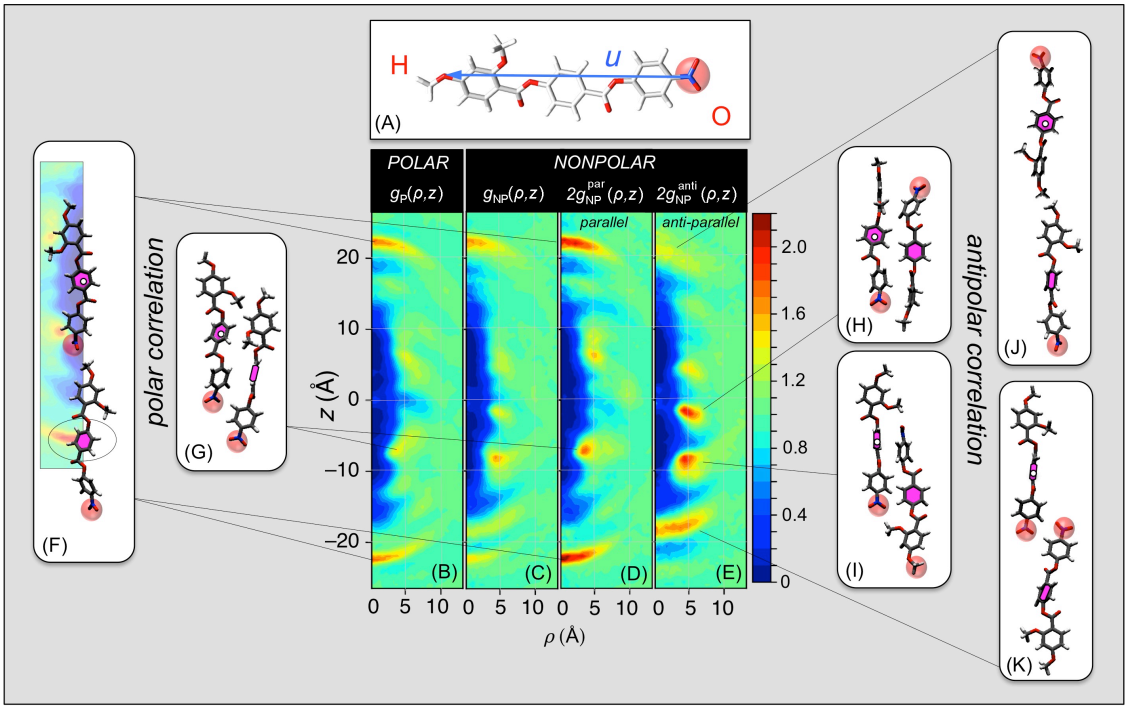

Figure S22: Results of atomistic molecular dynamic simulations designed specifically to explore the molecular interactions and resulting positional /orientational correlations responsible for the polar molecular ordering of RM734, shown in **(A)**. A nanoscale volume containing 384 molecules is equilibrated in these simulations into two distinct LC states: a *POLAR* system with all polar molecular long axes, ***u***, along +*z*, and a *NONPOLAR* system with half along +*z* and half along -*z*. Equilibration of the molecular conformation and packing is readily achieved but end-to-end flips are rare so the equilibrated states remain in the limit of polar or non-polar nematic order, respectively. (**B,F,G**) The *POL* simulation shows directly the dominant pair correlations adopted by molecules that are polar ordered, in the form of conditional probability densities, $g(\rho,z)$, of molecular centers (magenta) around a molecule with its center at the origin (white dot) and long axis ***u*** along *z*. The $g(\rho,z)$ are φ-averaged to be uniaxially symmetric, reflecting the uniaxial symmetry of the N and N_F phases. They exhibit a molecule-shaped, low-density region ($g(\rho,z) \sim 0$) around the origin resulting from the steric overlap exclusion of the molecules; an asymptotic constant value at large ρ giving the normalized average density ($g(\rho,z) = 1$); and distinct peaks indicating preferred modes of molecular packing. This analysis reveals two principal preferred packing modes in the *POL* system: (**B,F**) polar head-to-tail association stabilized by the attraction of the terminal nitro and methoxy groups, and (**B,G**) polar side-by-side association governed by group charges along the molecule, nitro-lateral methoxy attraction, and steric interactions of the lateral methoxys. (**D,E**) The *NONPOL* system exhibits distinct correlation functions for antiparallel and parallel molecular pairs, $g_{NP}{}^{anti}(\rho,z)$ and $g_{NP}{}^{par}(\rho,z)$. (**D,H,I**) The preferred antiparallel packing gives strong side-by-side correlations, governed by group charges along the molecule; and (**D,J,K**) weaker antipolar nitro-nitro end-to-end association. (**D,F,G**) The parallel correlations in the *NONPOL* system are the most relevant to the stability of polar order in the N_F phase, as they are determined by the inherent tendency of the molecular interactions for polar ordering in the presence of enforced polar disorder. Comparison of (**B**) and (**D**) shows identical preferred modes of parallel association in the two systems, with the *POL* system correlations being even stronger in the *NONPOL* system. This is clear evidence that the polar packing motifs giving the correlation functions (**B**) and (**D**), exemplified by the sample *POL* configurations (**F**) and (**G**), stabilize the polar order of the N_F.

Intermolecular correlations – In order to make headway in understanding the roles of molecular structure and interaction in RM734 and its relation to polar ordering, we applied the atomistic simulations to probe molecular association and packing in the *POL* and *NONPOL* systems. We characterized molecular pair positional and orientational correlations by measuring several $g(\rho,z)$, the conditional densities of molecular centers about a molecule fixed with its center at the origin, with its long axis ***u*** in ***Fig. S22A*** parallel to ***z*** and pointing in the +z direction [nitro group near (ρ,z) = (0,-10 Å) and the methoxy group near (ρ,z) = (0,10 Å)]. Here the center of the molecule is defined as the midpoint of ***u*** in ***Fig. S22A***, and the $g(\rho,z)$ are angular averages about ***z*** of density over the azimuthal orientations of the molecule at the origin. The $g(\rho,z)$ are therefore independent of azimuthal angle φ, reflecting uniaxial nematic symmetry. They all exhibit a correlation hole ($g(\rho,z)$ ~0) around the origin and extended along z for ~ 1.5 molecular lengths where other molecular centers are excluded because of steric repulsion and the strong nematic orientational ordering. The pair distributions $g_{P}(\rho,z)$ and $g_{NP}(\rho,z)$ for the *POL* and *NONPOL* systems, shown in ***Fig. S22B,*** and ***S22C-E***, respectively, display a number of striking features indicating specific molecular association motifs. In the *POL* system, pronounced arc-shaped peaks observed near (indicate a strong tendency for head-to-tail association of parallel pairs of molecules, as illustrated by the representative pair configuration shown in ***Fig. S22C***. Such head-to-tail configurations are characterized by close association of positively charged H atoms in the terminal methoxy group of the molecule at the origin with negatively charged O atoms in the nitro group of its neighbor, suggesting that head-to-tail association is in large part the result of specific electrostatic interactions. Prominent *off-axis* peaks near are also observed in $g_{P}(\rho,z)$, and analysis of pair configurations associated with these peaks (e.g., ***Fig. S22E***) reveals close association of oppositely charged atoms, including close contacts between positively charged H atoms in the lateral methoxy group and the negatively charged O atom in the terminal methoxy group, and between negatively charged O atoms in the nitro and carbonyl groups and positively charged H atoms in the phenyl rings. These observations also point to specific electrostatic interactions stabilizing polar pair configurations. In the *POLAR* simulation the lateral methoxys appear to be key to establishing the relative positioning of the side-by-side molecular associates that prefer polar ordering. The presence of a region of reduced probability ('correlation hole') in $g_{P}(\rho,z)$ near shows that side-by-side configurations of parallel pairs of molecules are relatively unfavorable, presumably due to the excluded volume of their lateral methoxy groups.

The *NONPOL* system forces both antiparallel and parallel molecular pairs, which give correlation functions, $g_{NP}{}^{anti}(\rho,z)$ and $g_{NP}{}^{par}(\rho,z)$, that exhibit very strongly expressed, polarity-dependent molecular recognition. In the on-axis or nearly on-axis peaks of $g_{NP}{}^{anti}(\rho,z)$ in ***Fig. S22G*** we observe that the z → -z symmetry of $g_{NP}{}^{anti}(\rho,z)$ is the most strongly broken, as expected since HO-OH association will be different from OH-HO association. Thus, there is a prominent arc-shaped peak near , arising from HO-OH antiparallel lateral association of terminal nitro groups, illustrated by the representative pair configuration in ***Figs. S22G,K***. Pair configurations associated with this peak are characterized by close contacts between negatively charged nitro O atoms and positively charged phenyl H atoms adjacent to the nitro group, suggesting that these configurations are electrostatically stabilized. In contrast, the OH-HO associations between terminal methoxy groups (associated with the peak near) are very weak (***Figs. S22G,J***).

The *NONPOL* system contribution to $g_{NP}{}^{anti}(\rho,z)$ from side-by-side antiparallel pairs (***Figs. S22G-I***), shows a prominent peak near , associated with pair configurations characterized by close contacts between positively charged H atoms in the lateral methoxy group and negatively charged nitro O atoms (an example of which is shown in ***Fig. S2I***). The side-by-side pair configurations associated with the peak near similarly involve close contacts between oppositely charged O and H atoms in the ester groups and phenyl rings. These findings provide further support for the hypothesis that electrostatic interactions between

specific oppositely charged atoms play a dominant role in stabilizing the characteristic pair configurations observed in our simulations.

The *NONPOL* system parallel pair correlation function $g_{NP}^{par}(\rho,z)$ in ***Fig. S22F*** is very similar to the *POL* system $g_P(\rho,z)$ in ***Fig. S22C,*** indicating a nanosegregation of the *par* and *anti* components, a mixture of OH-OH-OH and HO-HO-HO chains with the OH-HO at their interfaces. Remarkably, the polar features of $g_P(,z)$ are not only dominant in $g_{NP}^{par}(\rho,z)$ but even more pronounced than in $g_P(,z)$ itself. This suggests that there are certain polar associations in the *POL* system that can reduce the overall polar order, but that can be replaced by antipolar associations in the *NONPOL* system that are more favorable for nearby polar order. In any case, the simulations show that the polar correlations in ***Figs. S22B,C,F,E***, emergent in both *POL* and *NONPOL* systems, are the only packing motifs stabilizing polar order and therefore must be the principal drivers stabilizing the N_F phase.

The Plupolar Nematic – The *POL* simulation equilibrates a state in which end-to-end flipping is kinetically arrested and the periodic boundary conditions constrain the allowed wavelengths of orientation fluctuations to $\lambda_x < 55$ Å and $\lambda_z < 70$ Å. The remnant short ranged fluctuations create the pair correlations exhibited in ***Fig. S22***, which are confined to the volume $\rho < 10$ Å and $z < 30$ Å about the origin, molecular neighbor separation scales, well within the dimensions of the simulation box. These conditions create a *"plupolar" (plus quam pola*r [49]) equilibrium state of constrained polar ordering yielding the simulated P_S values in ***Fig. 3*** (open circles). Comparing these values with the RM734 data shows that, on the one hand, in the *plupolar* state the fluctuations that lead to the phase transition are clearly suppressed, while the remnant short range fluctuations give a P_S value exhibiting only a weak dependence on temperature. On the other hand, this P_S gives a good account of the polarization density of the N_F at low temperature, evidence that at low T the N_F phase approaches some comparable *plupolar*-like condition of having only short-range fluctuations, and that the simulated $g(\rho,z)$ are characterizing their remnant correlations. This state may be glassy, if the strong T-dependence of the viscosity is any indicator.

SECTION S11 – *MOVIE CAPTIONS*

Video 1: *Formation of domains with opposite polarization at the transition from the N to the N_F phase* – RM734 is filled in the isotropic phase into an 11 μm-thick cell with weak bidirectional planar surface rubbing. The sample is slowly cooled from N to N_F at -1ºC/min with no external field. The crossed analyzer and polarizer are oriented along the horizontal and vertical directions, and the rubbing is at 45º relative to the polarizer. As the transition is approached, a texture of stripe-like regions extended along the rubbing direction appears which, upon cooling through the transition, are identifiable as slender domains with opposite polarization. In the process of further cooling down, the texture coarsens into bigger domains with uniform polarization, including distinctive, lens-shaped domains embedded in large regions domains with the opposite polarization. The field response of two such lens-shaped domains, visible here in the top-middle part of the field of view, are shown (at higher magnification) in Video 2. Linear defects caused by inhomogeneities in the alignment layers are visible in both phases. The horizontal dimension is 712 μm. This video is speeded up by a factor 32x.

Video 2: *Electric field response of lens-shaped polarization reversal domains in the N_F phase* – RM734 is filled in the isotropic phase into an 11 μm-thick cell with weak bidirectional planar surface rubbing. The crossed polarizer and analyzer are oriented along the horizontal and vertical directions. A small DC. field of around 0.5 V/cm is applied in the plane of the sample, along the rubbing direction, which is at 45º to the polarizers. Since the applied field is opposed to the polarization inside each lens-shaped domain, electrical torques cause the director field in the cell interior to twist, changing the birefringence color inside the domains. When the applied field is sufficiently large, the surface anchoring is overcome by the electrical

torque. This resulting in domain wall movement that mediates polarization reversal at the surfaces, so that the polarization is eventually uniformly aligned along the field through the thickness of the cell and the original domain disappears. Outside the domain, on the other hand, the polarization is essentially already along the field so there is little change in the birefringence. The left domain remains visible because of higher surface anchoring energy in that part of cell. Near the end of the video, the field is reversed and the left domain becomes uniform, while with the region around it now becomes twisted in response to the electrical torques. The cell temperature is 120ºC and the horizontal dimension is 150 μm.

Video 3: *Giant ferro-electro-nemato-hydrodynamic flow and transport in N_F phase* – The sample liquid crystal is sandwiched between two glass substrates without surface treatment. The thickness of the cell is ~10 μm. The crossed polarizer and analyzer are oriented along the horizontal and vertical directions. A pair of square, gold electrodes are deposited onto one of the glass substrates such that there is a 60 μm gap between them. The electrodes are visible at the top of the field of view. When a 5 V, 0.1 Hz square-wave voltage is applied to the electrodes, a fringing field appears in the liquid crystal, with the electric field lines forming concentric circles centered around the gap. The sample is heated from the N_F to the N phase at +1ºC/min (the cell temperature is indicated on the video)., In the N_F phase, the sample shows huge flow effects, with the director orientation and the flow velocity both following the field lines, . Charged defects move back and forth with the flow, changing direction with the direction of flow depending on the sign of the applied field. In the N_F phase, the sample shows huge flow effects, with charged defects moving back and forth with the flow, while in the N phase, in contrast, there is little flow in response to the field. The first part of the video (at 126ºC) is in real time, the second part, while the cell is heated, is speeded up by a factor of 32x. The transition starts at around 130ºC (the cell temperature is indicated on the video). The horizontal dimension is 2.7 mm.

Video 4: *Electric field Response of polarization domains in the N_F phase during applied field reversal* – RM734 is filled in the isotropic phase into an 11 μm-thick cell with weak bidirectional planar surface rubbing. Two ITO electrodes deposited on the glass substrates with a gap of 1 mm are used to apply in-plane electric fields. The crossed polarizer and analyzer are aligned horizontally and vertically and the electric field applied is applied along the horizontal direction. The lower part of the cell is empty so this region, along with several circular air bubbles, appears completely dark under crossed polarizer and analyzer. The upper part of the cell is filled with RM734. At the start of the video, the polarization is aligned uniformly over much of the field of view by a small field of 2 V/cm. Since the director is aligned along the polarizer, the cell appears dark. After about 1 second, the sign of the applied field is reversed. The polarization field becomes orientationally unstable and develops a bend instability that leads to the originally homogeneous, large domain breaking up into many stripe-like domains in which the polarization rotates alternately clockwise and counter-clockwise. The domain walls of these domains form perpendicular to the applied field direction, thus avoiding splay of the polarization and reducing polarization space charge. A few seconds after the field is applied, the surface anchoring is overcome by the electrical torques and the director at the cell surfaces begins to reorient via domain wall movement, first at one surface then at the other. The birefringence color of the cell changes with the passage of each domain wall. Once both domain walls have passed by, we are left with a uniform region where the polarization is again aligned with applied field. After 15 seconds, before the entire cell has been reoriented by the applied field, the field direction is reversed again. The (black) uniform states become twisted and the (initially colorful) states that were twisted relax to become uniform. The cell temperature is 110ºC and the horizontal dimension is 712 μm.

Video 5: *Response of polarization domains in the NF phase to applied field reversal* – RM734 is filled in the isotropic phase into an 11 μm-thick cell with weak bidirectional planar surface rubbing. This video shows a

phenomenon similar to that in video 4 but at a larger magnification. Two ITO electrodes deposited on the glass substrates with a gap of 1 mm are used to apply in-plane electric field. The crossed polarizer and analyzer are aligned horizontally and vertically and the electric field is applied along the horizontal direction. At the start of the video, the polarization is uniformly aligned along the polarizer and appears dark. In the field of view, there is a single domain except for several surface defect lines and air bubbles.

After about 2s, an increasing electric field with opposite direction is applied. The polarization field becomes orientationally unstable and develops a bend instability. The birefringence color of those domains changes gradually due to the increasingly distorted twist structure. Finally, the surface anchoring is overcome by the electrical torques and the cell surfaces begins to reorient via domain wall movement. The two separate wall movements are more obvious in this magnified video. We are left with a uniform domain aligned with field once again when both walls passed by. After 10 seconds, the field is reversed again. A similar polarization reversal process occurs. The cell temperature is 110ºC and the horizontal dimension is 259 μm.

SUPPLEMENTARY INFORMATION REFERENCES

1 R. J. Mandle, S. J. Cowling, and J. W. Goodby, A nematic to nematic transformation exhibited by a rod-like liquid crystal. *Phys. Chem. Chem. Phys.* **19**, 11429-11435 (2017).

2 R. J. Mandle, S. J. Cowling, and J. W. Goodby, Rational design of rod-like liquid crystals exhibiting two nematic phases. *Chemistry – a European Journal* **23**, 14554-14562 (2017).

3 P. Oswald, P. Pieranski, *Nematic and Cholesteric Liquid Crystals* (Taylor & Francis, Boca Raton, 2005).

4 M. A. Handschy, N. A. Clark, Structures and responses of ferroelectric liquid crystals in the surface-stabilized geometry. *Ferroelectrics* **59**, 69-116 (1984).

5 R. Pindak, C. Y. Young, R. B. Meyer, N. A. Clark, Macroscopic orientation patterns in smectic-C films. *Physical Review Letters* **45**, 1193-1196 (1980).

6 R. B. Meyer, L. Liébert, L. Strzelecki, P. Keller, Ferroelectric liquid crystals. *J. Phys.* **36**, L-69 (1975).

7 C. Y. Young, R. Pindak, N. A. Clark, R. B. Meyer, Light-scattering study of two-dimensional molecular-orientation fluctuations in a freely suspended ferroelectric liquid-crystal film. *Physical Review Letters* **40**, 773 (1978).

8 M. H. Lu, K. A. Crandall, C. Rosenblatt, Polarization-induced renormalization of the B_1 elastic-modulus in a ferroelectric liquid-crystal. *Physical Review Letters* **68**, 3575-3578 (1992).

9 M. H. Lu, K. A. Crandall, C. Rosenblatt, R. G. Petschek, Ion-director coupling in a ferroelectric liquid-crystal. *Physical Review E* **47**, 1139-1143 (1993).

10 R. A. Pelcovits, R. B. Meyer, J.-B. Lee, Dynamics of the molecular orientation field coupled to ions in two-dimensional ferroelectric liquid crystals. *Physical Review E* **76**, 021704 (2007).

11 N. A. Clark, D. Coleman, J. E. Maclennan, Electrostatics and the electro-optic behavior of chiral smectics C: 'block' polarization screening of applied voltage and 'V-shaped' switching. *Liquid Crystals* **27**, 985–990 (2000).

12 D. Coleman, D. Mueller, N. A. Clark, J. E. Maclennan, R. F. Shao, S. Bardon, D. M. Walba, Control of molecular orientation in electrostatically stabilized ferroelectric liquid crystals. *Physical Review Letters* **91**, 175505 (2003).

13 Z. Zhuang, J.E. Maclennan, N.A. Clark, Device applications of ferroelectric liquid crystals: importance of polarization charge interactions. *Proceedings of the Society of Photo-Optical Instrumentation Engineers* **1080**, 110-114 (1989).

14 M. A. Handschy, N. A. Clark, Stroboscopic microscopy of fast electro-optic switching in a ferroelectric smectic C liquid crystal. *Applied Physics Letters* **41**, 39-41 (1982).

15 Y. Shen, T. Gong, R. Shao, E. Korblova, J. E. Maclennan, D. M. Walba, N. A. Clark, Effective conductivity due to continuous polarization reorientation in fluid ferroelectrics. *Physical Review E* **84**, 020701(R) (2011).

16 R. J. Mandle, A. Mertelj, Orientational order in the splay nematic ground state. *Phys. Chem. Chem. Phys.* **21**, 18769-18772 (2019).

17 A. Mertelj, L. Cmok, N. Sebastian, R. J. Mandle, R. R. Parker, A. C. Whitwood, J. W. Goodby, M. Copic, Splay nematic phase. *Phys. Rev. X* **8**, 041025 (2018).

18 N. Sebastian, L. Cmok, R. J. Mandle, M. Rosario de la Fuente, I. Drevenšek Olenik, M. Copic, A. Mertelj, Ferroelectric-ferroelastic phase transition in a nematic liquid crystal. *Physical Review Letters* **124**, 037801 (2020).

19 G. A. Hinshaw, Jr., R. G. Petschek, R. A. Pelcovits, Modulated phases in thin ferroelectric liquid-crystal films. *Physical Review Letters* **60**, 1864 –1868 (1988).

20 J. Als-Nielsen, R. J. Birgeneau, Mean field theory, the Ginzburg criterion, and marginal dimensionality of phase transitions. *American Journal of Physics* **45**, 554-560 (1977).

21 M. Copic, A. Mertelj, Q-tensor model of twist-bend and splay nematic phases. *https://arxiv.org/pdf/1910.01424.pdf*

22 J. Kotzler, Critical phenomena in dipolar magnets. *Journal of Magnetism and Magnetic Materials* **54-57**, 649-654 (1986).

23 J. Als-Nielsen, Experimental test of renormalization group theory on the uniaxial, dipolar coupled ferromagnet $LiTbF_4$. *Physical Review Letters* **37**, 1161-1164 (1976).

24 J. Als-Nielsen, R. J. Birgeneau, Mean field theory, the Ginzburg criterion, and marginal dimensionality of phase transitions. *American Journal of Physics* **45**, 554-560 (1977).

25 A. Aharony, M. E. Fisher, Critical behavior of magnets with dipolar interactions. 1. Renormalization group near 4 dimensions. *Physical Review B* **8**, 3323-3341 (1973).

26 A. Aharony, Critical behavior of magnets with dipolar interactions. 5. Uniaxial magnets in d-dimensions. *Physical Review B* **8**, 3363-3370 (1973).

27 G. Ahlers, A. Kornblit, H. J. Guggenheim, Logarithmic corrections to the Landau specific heat near the Curie temperature of the dipolar Ising ferromagnet LiTbF4. *Physical Review Letters* **34**, 1227-1230 (1975).

28 N.A. Clark, Surface memory effects in liquid crystals: influence of surface composition. *Physical Review Letters* **55**, 292-295 (1985).

29 R. Barberi, F. Ciuchi, G.E. Durand, M. Iovane, D. Sikharulidze, A. M. Sonnet, E. G. Virga, Electric field induced order reconstruction in a nematic cell. *European Physical Journal E* **1**, 61-71 (2004).

30 R. Barberi, F. Ciuchi, F G. Lombardo, R. Bartolino, G. E. Durand, Time resolved experimental analysis of the electric field induced biaxial order reconstruction in nematics. *Physical Review Letters* **93**, 137801 (2004).

31 J. V. Selinger, Z. G. Wang, R. F. Bruinsma, C. M. Knobler, Chiral symmetry breaking in Langmuir monolayers and smectic films. *Physical Review Letters* **70**, 1139-1142 (1993).

32 M. A. Handschy, N. A. Clark, Structures and responses of ferroelectric liquid crystals in the surface-stabilized geometry. *Ferroelectrics* **59**, 69-116 (1984).

33 D. R. Link, G. Natale, R. F. Shao, J. E. Maclennan, N. A. Clark, E. Körblova, D. M. Walba, Spontaneous formation of macroscopic chiral domains in a fluid smectic phase of achiral molecules. *Science* **278**, 1924-1927 (1997).

34 D. A. Coleman, J. Fernsler, N. Chattham, M. Nakata,Y. Takanishi, E. Körblova D. R. Link, R.-F. Shao, W. G. Jang, J. E. Maclennan, O. Mondainn-Monval, C. Boyer, W. Weissflog, G. Pelzl, L.-C. Chien, D.M. Walba, J. Zasadzinski, J Watanabe, H. Takezoe, N. A. Clark, Polarization modulated smectic liquid crystal phases. *Science* **301**, 1204-1211 (2003).

35 R. D. Kamien, J. V. Selinger, Order and frustration in chiral liquid crystals. *J. Phys.: Condens. Matter* **13**, R1–R22 (2001).

36 R. B. Meyer, Piezoelectric effects in liquid crystals. *Physical Review Letters* **22**, 918-921 (1969).

37 M. Magnus, *Michel-Levy Color Chart* (Carl Zeiss Microscopy GmbH, 07745 Jena, Germany, 2011) https://www.zeiss.com/microscopy. Retrieved 16 October 2020.

38 N. A. Clark, D. Coleman, J. E. Maclennan, Electrostatics and the electro-optic behavior of chiral smectics C: 'block' polarization screening of applied voltage and 'V-shaped' switching. *Liquid Crystals* **27**, 985–990 (2000).

39 D. Coleman, D. Mueller, N. A. Clark, J. E. Maclennan, R. F. Shao, S. Bardon, D. M. Walba, Control of molecular orientation in electrostatically stabilized ferroelectric liquid crystals. *Physical Review Letters* **91**, 175505 (2003).

40 O. Borodin, Polarizable force field development and molecular dynamics simulations of ionic liquids. *J. Phys. Chem.* B **113**, 11463–11478 (2009).

41 D. Bedrov, et al., Polarizable molecular dynamics simulations of ionic and polar systems. *Chem. Rev.* **119**, 7940–7995 (2019).

42 M. J. Frisch, M. Head-Gordon, J. A. Pople, A direct MP2 gradient method. *Chem. Phys. Lett.* **166**, 275–280 (1990).

43 R. A. Kendall, T. H. Dunning, R. J. Harrison, Electron affinities of the first-row atoms revisited. Systematic basis sets and wave functions. *J. Chem. Phys.* **96**, 6796–6806 (1992).

44 Y. Zhao, N. E. Schultz, D. G. Truhlar, Design of density functionals by combining the method of constraint satisfaction with parametrization for thermochemistry, thermochemical kinetics, and noncovlent interactions. *J. Chem. Theory Comput.* **2**, 364–382 (2006).

45 B. J. Palmer, Direct application of SHAKE to the velocity Verlet algorithm. *J. Comput. Phys.* **104**, 470–472 (1993).

46 I. C. Yeh, M. L. Berkowitz, Ewald summation for systems with slab geometry. *J. Chem. Phys.* **111**, 3155–3162 (1999).

47 B. T. Thole, Molecular polarizabilities calculated with a modified dipole interaction. *Chem. Phys.* **59**, 341–350 (1981).

48 D. J. Evans, B. L. Holian, The Nose–Hoover thermostat. *J. Chem. Phys.* **83**, 4069–4074 (1985).

49 https://en.wikipedia.org/wiki/Pluperfect. Retrieved 14 February 2020.
